# Tumor suppressor DRD2 facilitates M1 macrophages and restricts NF-κB signaling to trigger pyroptosis in breast cancer

**DOI:** 10.7150/thno.58322

**Published:** 2021-03-05

**Authors:** Yiqing Tan, Ran Sun, Lei Liu, Dejuan Yang, Qin Xiang, Li Li, Jun Tang, Zhu Qiu, Weiyan Peng, Yuanyuan Wang, Lin Ye, Guosheng Ren, Tingxiu Xiang

**Affiliations:** 1 Key Laboratory of Molecular Oncology and Epigenetics, The First Affiliated Hospital of Chongqing Medical University, Chongqing, China.; 2 Department of Endocrine and Breast Surgery, The First Affiliated Hospital of Chongqing Medical University, Chongqing 400016, China.; 3 Chongqing Jiulongpo People's Hospital, Chongqing 400024, China.

**Keywords:** DRD2, Breast Cancer, Tumor Suppressor Genes, Macrophages, Pyroptosis.

## Abstract

**Rationale:** Breast cancer (BrCa) is the most common cancer worldwide, and the 5-year relative survival rate has declined in patients diagnosed at stage IV. Advanced BrCa is considered as incurable, which still lack effective treatment strategies. Identifying and characterizing new tumor suppression genes is important to establish effective prognostic biomarkers or therapeutic targets for late-stage BrCa.

**Methods:** RNA-seq was applied in BrCa tissues and normal breast tissues. Through analyzing differentially expressed genes, DRD2 was selected for further analysis. And expression and promoter methylation status of DRD2 were also determined. DRD2 functions were analyzed by various cell biology assays *in vitro*. Subcutaneous tumor model was used to explore DRD2 effects *in vivo*. A co-cultivated system was constructed to investigate interactions of DRD2 and macrophages *in vitro*. WB, IHC, IF, TUNEL, qRT-PCR, Co-IP, Antibody Array, and Mass Spectrum analysis were further applied to determine the detailed mechanism.

**Results:** In BrCa, DRD2 was found to be downregulated due to promoter methylation. Higher expression of DRD2 positively correlated with longer survival times especially in HER2-positive patients. DRD2 also promoted BrCa cells sensitivity to Paclitaxel. Ectopic expression of DRD2 significantly inhibited BrCa tumorigenesis. DRD2 also induced apoptosis as well as necroptosis *in vitro* and *in vivo*. DRD2 restricted NF-κB signaling pathway activation through interacting with β-arrestin2, DDX5 and eEF1A2. Interestingly, DRD2 also regulated microenvironment as it facilitated M1 polarization of macrophages, and triggered GSDME-executed pyroptosis.

**Conclusion:** Collectively, this study novelly manifests the role of DRD2 in suppressing BrCa tumorigenesis, predicting prognosis and treatment response. And this study further reveals the critical role of DRD2 in educating M1 macrophages, restricting NF-κB signaling pathway and triggering different processes of programmed cell death in BrCa. Taking together, those findings represent a predictive and therapeutic target for BrCa.

## Introduction

Breast cancer (BrCa) is the most common cancer and the second leading cause of cancer-related death in female. With the development of mammography screening, the 5-year relative survival rate has been increased, but treatment of recurrent and metastatic BrCa is still a great challenge 1, 2. It is therefore important to understand the molecular mechanism underlying BrCa progression and identify new and effective prognostic biomarkers or therapeutic targets for BrCa.

Tumor suppression genes (TSGs) are critical for tumor progression, which are often downregulated and frequently silenced by DNA methylation in BrCa 3, 4. DNA methylation is the major epigenetic modification 5. The expression of TSGs silenced by methylation could be restored by DNA methyltransferase inhibitors such as 5'-Aza-deoxycytidine (Aza). This has already been used in clinical practice 6. This study aimed to identify and characterize a possible new TSG related to BrCa prognosis and treatment response, which could further facilitate comprehensive treatment of BrCa. Through analyzing the gene profile of RNA sequences (RNA-seq) generated from BrCa and normal breast tissues, *DRD2* was selected, and further experiments confirmed its downregulated expression as well as hypermethylated status in BrCa.

DRD2 (D2 dopamine receptor) belongs to dopamine receptors family, and is a dominated member of D2-like receptors. Dopaminergic signaling is critical in the nervous system and is involved in working memory, reward, learning and so on. Besides, accumulating evidences have indicated that DA also plays a significant physiological role in the immune system and carcinoma development 7. DA has been reported to inhibit tumor growth and metastasis by activating DA receptors 8, 9. Moreover, DA receptor regulates tumor development by participating in reprogramming the tumor-associated microenvironment (TME) 10. DA also weakens EGF-induced EGFR activation 11. However, whether and how DRD2 mediates BrCa progression remains largely unknown.

In this study, *DRD2* was found to be downregulated due to promoter methylation. DRD2 also promoted BrCa patients' survival times and drug sensitivity to Paclitaxel (PTX). DRD2 was more common in HER2-negative patients than in HER2-positive patients, and DRD2 also downregulated EGFR and HER2 expression. DRD2 functioned as a TSG and induced apoptosis as well as necroptosis in BrCa cells. DRD2 also educated macrophages (Mφ) to M1 phenotype and induced GSDME-executed pyroptosis during crosstalk. However, interestingly, mechanistic investigations revealed that DRD2 restricted NF-κB signaling activation by activating β-arrestin2, and downregulating DDX5 as well as eEF1A2. These events finally suppressed the initiation of NF-κB signaling pathway and phosphorylation of p65. Taken together, this study newly manifested the regulatory mechanisms of DRD2-mediated tumor suppressive effects and may contribute to the improvement of DRD2-based prognosis prediction and anticancer therapy.

## Results

### *DRD2* is transcriptionally downregulated through promoter methylation in BrCa

**Figure 1:**
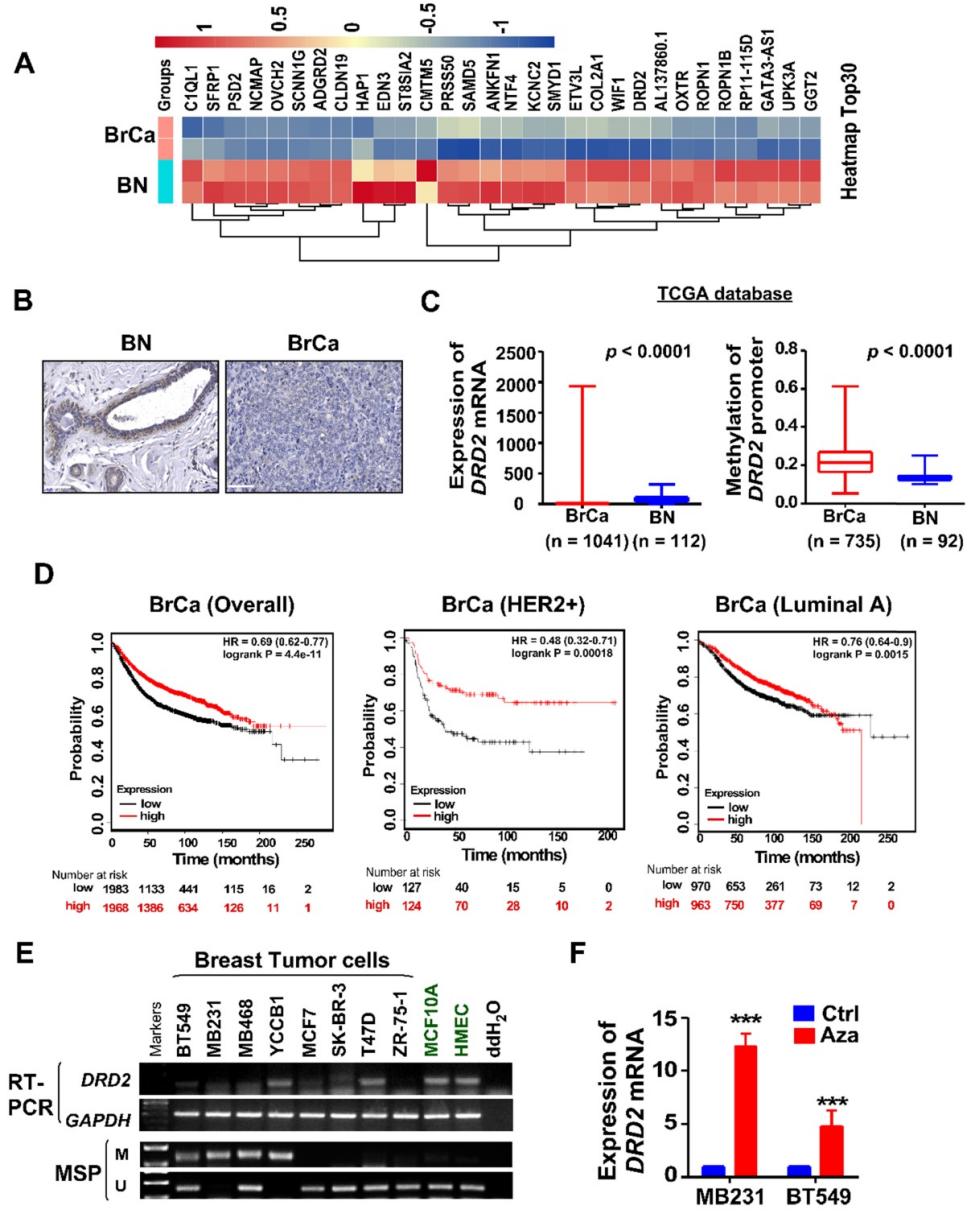
** DRD2 is transcriptionally downregulated through promoter methylation in BrCa.** (**A**) Heatmap of top 30 differentially expressed genes based on RNA-seq. Tissues derived from nor mal breast tissues and BrCa tissues were applied for RNA-seq analysis. Log2FC transformed and normalized values were used. (**B**) IHC staining of DRD2 in normal breast tissues and BrCa tissues. Bars, 60 μm. (**C**) Expression and methylation of *DRD2* based on TCGA database. Data are presented as mean ± SD; *p*-value was calculated using two-tailed Student's t test. *p* < 0.0001. (**D**) Online database Kmplot was used to analyze the effects of *DRD2* on the overall prognosis (left) of BrCa patients, the survival times of BrCa patients featured HER2-positive (middle), and the survival times of BrCa patients featured Luminal A (right). (**E**) mRNA expression (RT-PCR) and promoter methylation (MSP) analysis of *DRD2* in BrCa cells lines and mammary epithelial cell lines were all detected. (**F**) mRNA expression of *DRD2* after Aza treatment was determined by qRT-PCR. MDA-MB231 and BT549 were treated with Aza for 3 d. BrCa cells without Aza treatment were used as controls. Data are presented as mean ± SD; *p*-value was calculated using two-tailed Student's t test. ***, *p* < 0.001. BN, normal breast; BrCa: Breast cancer; Ctrl, Control; MSP, methylation-specific PCR; M, methylated; U, unmethylated.

To investigate novel potential TSGs, BrCa tissues and normal tissues were employed for RNA-seq screening. mRNA expression of *DRD2* was found to be remarkably downregulated in BrCa tissues compared with normal breast tissues **(Figure 1A)**. And higher protein level of DRD2 was also found in normal breast tissues compared with BrCa tissues by IHC staining **(Figure 1B)**. Consistently, downregulation of *DRD2* mRNA was also observed in BrCa based on a larger cohort from TCGA, and promoter methylation of *DRD2* was more frequent in BrCa as well **(Figure 1C)**. Expression and promoter methylation status were further analyzed on another online database MethHC. Unlike DRD3 and DRD4, DRD2 was found to be downregulated accompanying by high methylation status of promoter **(Figure S1A-C)**. The relationship between *DRD2* expression and pathological features in BrCa patients was analyzed based on TCGA database. The higher expression of *DRD2* showed a negative correlation with the patients' age **(Table 1)**. In addition, *DRD2* expression was higher in HER2-negative patients **(Table 1)**. The relationship between *DRD2* promoter methylation and pathological features was also analyzed. Results showed that increased methylation of *DRD2* was more common in older populations **(Table 2)**. Based on *Kmplot*, higher expression of *DRD2* promoted longer survival of BrCa patients, which was also seen in patients with the HER2-positive genotype. But this superiority was not seen in Luminal A patients **(Figure 1D)**. Downregulation or loss of mRNA expression along with promoter methylation of *DRD2* could be seen in almost all BrCa cell lines as compared with immortalized normal breast cell lines according to RT-PCR and MSP results **(Figure 1E)**. Thus, the high methylating frequency of *DRD2* might contribute to its downregulation BrCa. Supportively, pharmacologic demethylation with Aza restored the expression of *DRD2* in two silenced BrCa cell lines, MDA-MB231 and BT549 **(Figure 1F)**. In general, *DRD2* expression is silenced by promoter methylation in BrCa. DRD2 also significantly promotes prognosis of HER2-positive BrCa patients. DRD2 is a potential TSG and a promising biomarker to predict the prognosis of BrCa patients.

### DRD2 functions as a TSG and inhibits epithelial-mesenchymal transition (EMT)

**Figure 2:**
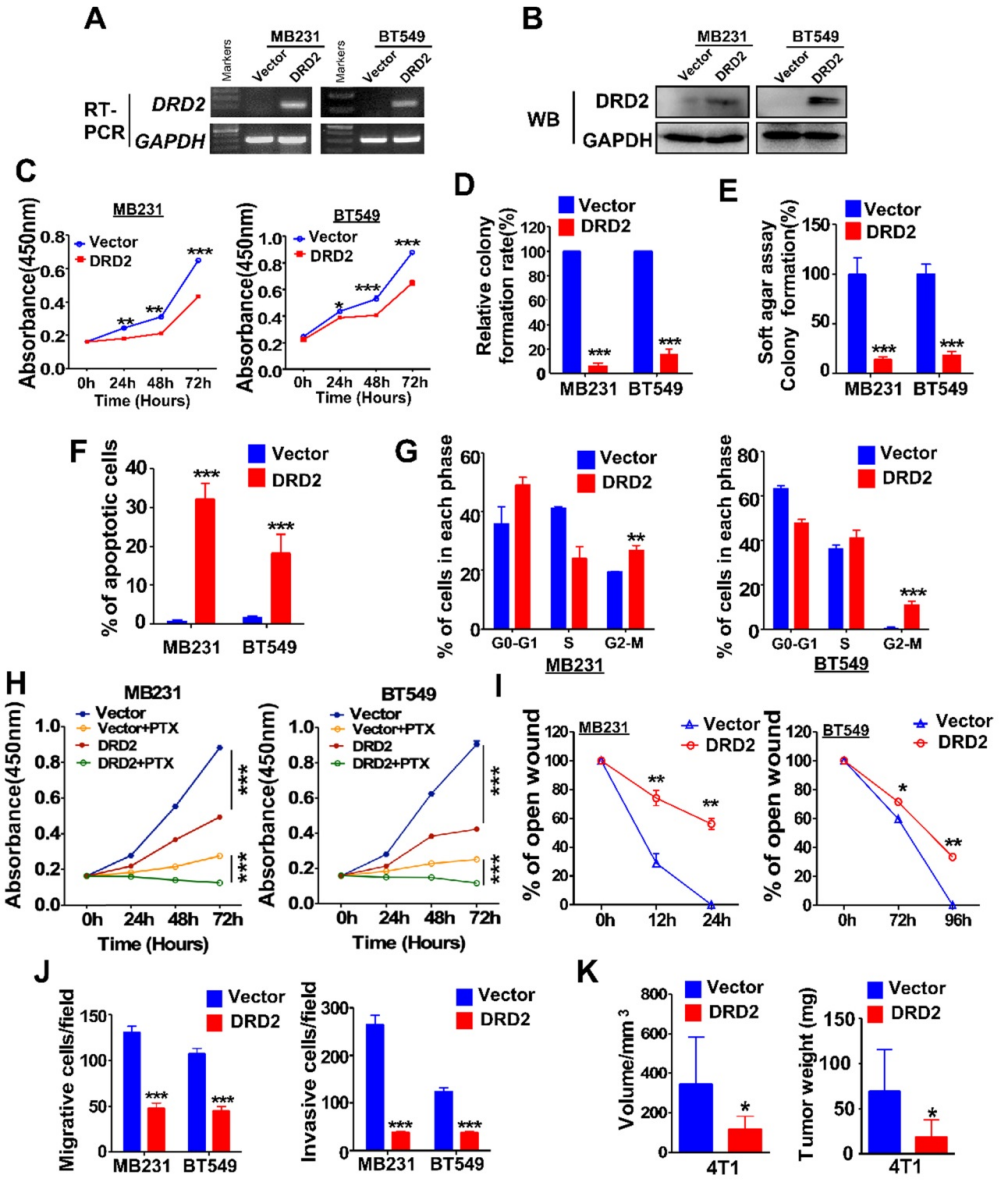
** DRD2 expression inhibits BrCa cells tumorigenesis *in vitro* and *in vivo*.** (**A** and **B**) Confirming ectopic *DRD2* mRNA expression by RT-PCR (A) and protein expression by WB (B). (**C**) Measurement of proliferation in Vector- and DRD2-transfected BrCa cells by CCK8 assay. (**D** and **E**) Histogram statistics of colony formation (D) and soft agar formation assay (E) to determine proliferation rates. (**F**) Histogram statistics showing analysis of apoptosis determined by AO/EB assay. (**G**) Histogram statistics of cell cycle distribution by FC. (**H**) Histogram statistics of proliferation rates in BrCa cells. CCK8 was performed to analyze effect of 891 DRD2 expression on chemosensitivity of BrCa cells to PTX. DMSO was used as controls. (**I** and **J**) Histogram showing effects of DRD2 on metastatic abilities in wound-healing (I) and Transwell® assay (J). Transwell® coated without (left) or with (right) Matrigel were applied to detecting migrative or invasive abilities of BrCa cells. (**K**) The volume and weight measurements of subcutaneous tumor model in BALB/c mice (8 mice per group). Volume = length × width2 × 0.5. DRD2/4T1 cells were used, and vector-transfected 4T1 cells were used as controls. Data are presented as mean ± SD; P-value was calculated using two-tailed Student's t test. *, *p* < 0.05; **, *p* < 0.01; ***, *p* < 0.001. PTX, Paclitaxel. AO/EB, acridine orange/ethidium bromide.

MDA-MB231 and BT549 cells with stably overexpression of DRD2 were constructed and validated by RT-PCR **(Figure 2A)** and WB **(Figure 2B)**. Ectopic expression of DRD2 inhibited tumor cell growth as evidenced by CCK8 assay **(Figure 2C)**. And DRD2 also impaired survival capacity as shown in monolayer colony formation assay **(Figure 2D and Figure S2A)** and soft agar formation assay **(Figure 2E and Figure S2B)**. And apoptosis and cell cycle distribution assays were further performed. DRD2 expression significantly promoted BrCa cell apoptosis **(Figure 2F and Figure S2C)** and blocked both MDA-MB231 and BT549 in G2/M phase **(Figure 2G and Figure S2D)**. Besides, overexpression of DRD2 promoted BrCa cells sensitivity to PTX **(Figure 2H)**. Ectopic expression of DRD2 exhibited less capability of closing artificial wounds than the controls in the wound-healing assay **(Figure 2I and Figure S2E)**. In accordance with that, overexpression of DRD2 significantly decreased BrCa migration and invasion, as indicated in the Transwell® assay without or with coated Matrigel **(Figure 2J and Figure S2F-G)**. The tumor-suppressive effects of DRD2 were further determined *in vivo*. The subcutaneous tumor model indicated the reduction in both volume and weight of tumors derived from DRD2-overexpressing model **(Figure 2K and Figure S2H)**. Knocking down DRD2 in YCCB1, which expressed relatively higher DRD2, promoted proliferation and inhibited apoptosis **(Figure S2I-J)**. And downregulated expression of DRD2 also promoted metastatic ability of YCCB1 **(Figure S2K)**.

EMT is important in migration and invasion of cancer cells. The morphology of MDA-MB231 and BT549 changed to an epithelium-like type following DRD2 restoration **(Figure S3A)**. WB results indicated increased expression of E-cadherin and decreased expression of Vimentin as well as another pro-EMT transcription factor, ZEB1 **(Figure S3B)**. IF staining also indicated that ectopic expression of DRD2 induced MDA-MB231 to lose the mesenchymal marker Vimentin and acquire the epithelial marker E-cadherin **(Figure S3C)**. Taken together, DRD2 is capable of suppressing tumorigenesis *in vitro* and *in vivo*, and DRD2 also suppresses EMT of BrCa cells.

### DRD2-transfected BrCa facilitates M1 phenotype Mφ

**Figure 3:**
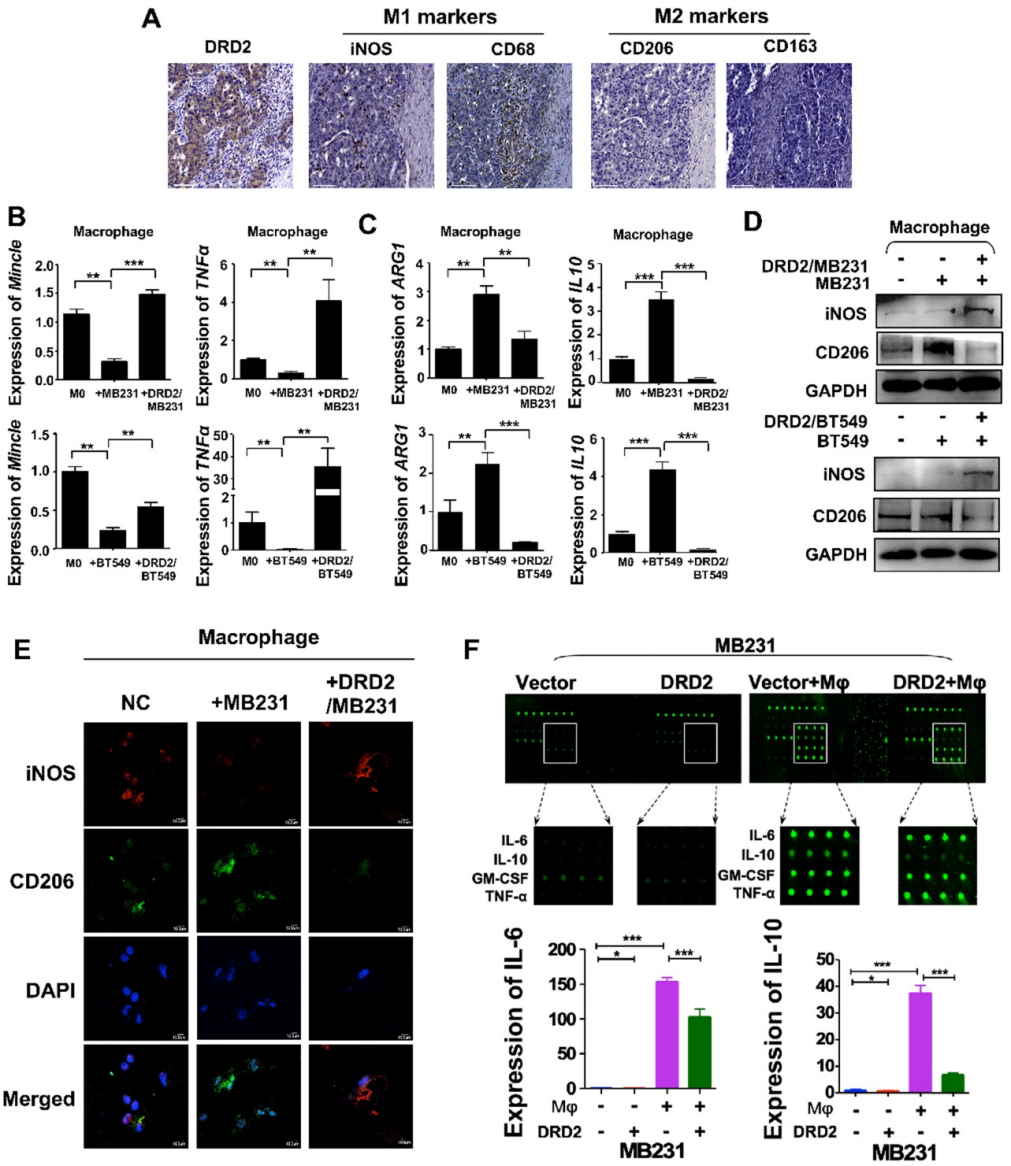
** DRD2 reprograms Mφ towards M1 phenotype and downregulates IL-6 and IL-10.** (**A**) IHC staining on serial sections of tissues from patients with BrCa. M1 markers, iNOS and CD68; M2 markers, CD206 and CD163. Bars, 80 μm. (**B** and** C**) qRT-PCR was used to detect M1 and M2 Mφ markers after co-cultivation with BrCa cells. Transwell® was applied to construct the co-culture system. The THP1- derived Mφ co-cultured with vector- and DRD2-transfected BrCa cells for 3 d. And primary THP-1-derived Mφ (M0) was used as control. (**D**) WB results of M1 and M2 markers after co-cultivation. (**E**) IF staining of M1 and M2 markers after co-cultivation. And THP1 cells were seeded in glass coverslips when differentiating to Mφ by PMA. M1 marker, iNOS; M2 marker, CD206. (**F**) An antibody array was used for cytokines detection of medium from BrCa cells. The medium used for detection was harvested after another 24 h incubation when finishing 3 d co-cultivation. Medium derived from BrCa cells was used as control. Fluorescence imaging (upper) and analysis of extracted data (lower) were shown. Data are presented as mean ± SD; P-value was calculated using two-tailed Student's t test. *, *p* < 0.05; **, *p* < 0.01; ***, *p* < 0.001.

DRD2 was reported to regulate polarization of Mφ to M1 previously 10. In this study, IHC results from BrCa patients indicated an increased infiltration of M1 Mφ and decreased filtration of M2 Mφ in BrCa tissues with higher DRD2 expression **(Figure 3A)**. To further investigate the function of DRD2 in regulating TAMs, a co-cultivated system of BrCa and Mφ was constructed. When Mφ was co-cultured with DRD2-expressing BrCa cells, markers of M1 phenotype were increased and M2 Mφ markers were downregulated as indicated by qRT-PCR **(Figure 3B-C)**. qRT-PCR also confirmed that vector-transfected BrCa cells regulated Mφ to M2 type **(Figure 3C)** 12. WB results determined that M1 marker iNOS was upregulated and M2 marker CD206 was downregulated by DRD2-expressing BrCa cells **(Figure 3D)**. And IF staining showed that Mφ exhibited M1 phenotype after co-cultivation with DRD2-expressing tumor cell **(Figure 3E)**. The results described above indicate that DRD2 in BrCa has the ability to reprogram Mφ to M1 phenotype.

To explore the key regulators polarizing Mφ towards M1 phenotype, a cytokine array analysis was performed. The results demonstrated that TNFα and IFN γ, two classical cytokines inducing M1 polarization, were not increased. However, IL-6 and IL-10 were downregulated dramatically by DRD2 after co-cultured with Mφ **(Figure 3F)**. The fluorescence value was analyzed and the downregulated of IL-6 and IL-10 were further confirmed **(Figure 3F)**. Thus, DRD2 could reprogram Mφ to M1 phenotype and significantly downregulate IL-6 and IL-10 during crosstalk.

### DRD2-reprogrammed Mφ induces pyroptosis in tumor cells

**Figure 4:**
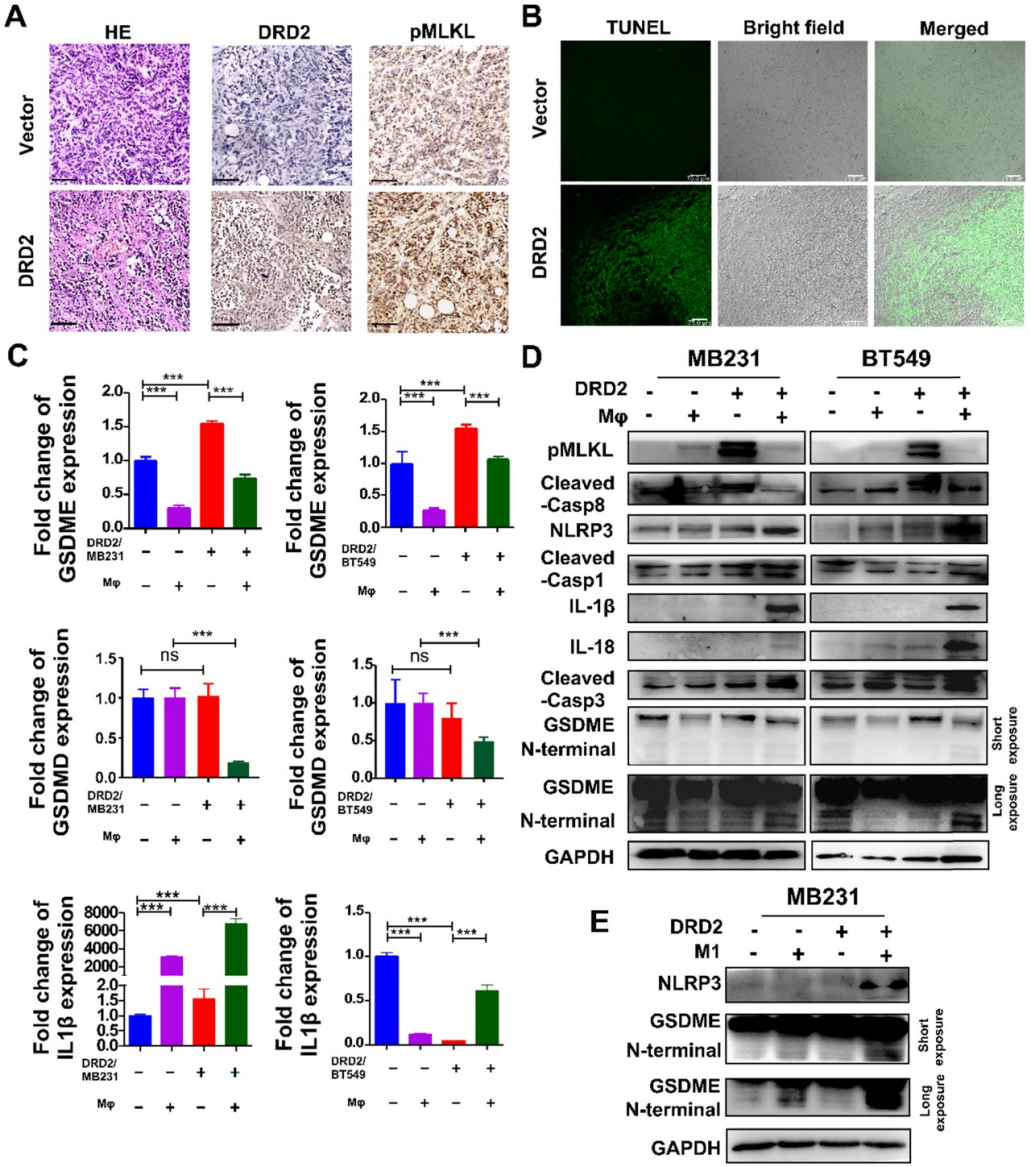
** DRD2 triggers pyroptosis during crosstalk with Mφ.** (**A** and **B**) Murinebreast cancer cell 4T1 used to construct subcutaneous tumor model. HE (A, left), IHC (A, middle and right) and TUNEL (B) assays were performed in samples derived from subcutaneous tumor model. Bars, 80 μm in (A); Bars, 75 μm in (B). (**C** and **D**) Pyroptosis markers were examined by qRT-PCR (C), and necroptosis, apoptosis, as well as pyroptosis markers were detected by WB (D) in vector- and DRD2-transfected BrCa cells co-cultivated with Mφ. BrCa cells cultured alone were used as controls. Data are presented as mean ± SD from biological replicates. P-value was calculated using two-tailed Student's t test. ***, *p* < 0.001. ns: not significant. (**E**) Markers of inflammasome (NLRP3) and pyroptosis (GSDME) were detected by WB. M1 Mφ was induced by LPS (200 ng/ml, 3 d). Medium derived from M1 Mφ was filtrated and applied to incubate Vector- and DRD2-MB231 for 3 d. Tumor cells cultured alone were used as control.

Tumor bearing DRD2 derived from the mice model exhibited increased tumor cell death as shown in HE **(Figure 4A)**. In IHC staining, DRD2-expressing 4T1 tumor samples exited higher expression of pMLKL **(Figure 4A)**, the executioner of necroptosis 13. DRD2 also promoted apoptosis *in vivo* according to TUNEL assay **(Figure 4B)**. GSDME instead of GSDMD was upregulated by DRD2 **(Figure 4C)**. Mφ further promoted GSDME expression in DRD2-transfected BrCa cells during crosstalk **(Figure 4C)**. And IL-1β was also upregulated by Mφ in DRD2-expressing BrCa cells **(Figure 4C)**. As WB results suggested, DRD2 induced the phosphorylation of MLKL, which was inhibited by Mφ during co-cultivation **(Figure 4D)**. Furthermore, DRD2 expression activated caspase-8, and the activation was inhibited by Mφ as a result of the co-cultivation **(Figure 4D)**. Hypothetically, DRD2 might induce pyroptosis during crosstalk with Mφ. The assembly of NLRP3 was triggered in DRD2-expressing BrCa cells when co-culturing with Mφ in BrCa cells. The maturation of IL-1β and IL-18 were also induced by activated caspase-1 proteolytically **(Figure 4D)** 14. Cleaved caspase-3 was upregulated during co-culture **(Figure 4D)**. Cleaved caspase-3 can cleave N-terminal of GSDME 15. In addition, N-terminal of GSDME was found to be cleaved in BrCa cells with DRD2 expression during co-cultivation **(Figure 4D)**. To determine whether the pyroptosis was triggered by M1 Mφ, the medium of LPS-induced M1 Mφ was used and results suggested that M1 Mφ only triggered the NLRP3 assembly and cleaved GSDME in BrCa cells with DRD2 expression **(Figure 4E)**. Results above indicate that DRD2 can induce programmed cell death (PCD) including apoptosis and necroptosis 16, and these events were switched to pyroptosis by Mφ. And M1 Mφ triggers pyroptosis of BrCa cells in a DRD2-dependent manner.

### DRD2 restricts NF-κB signaling activation by interrupting phosphorylation of TAK1

**Figure 5:**
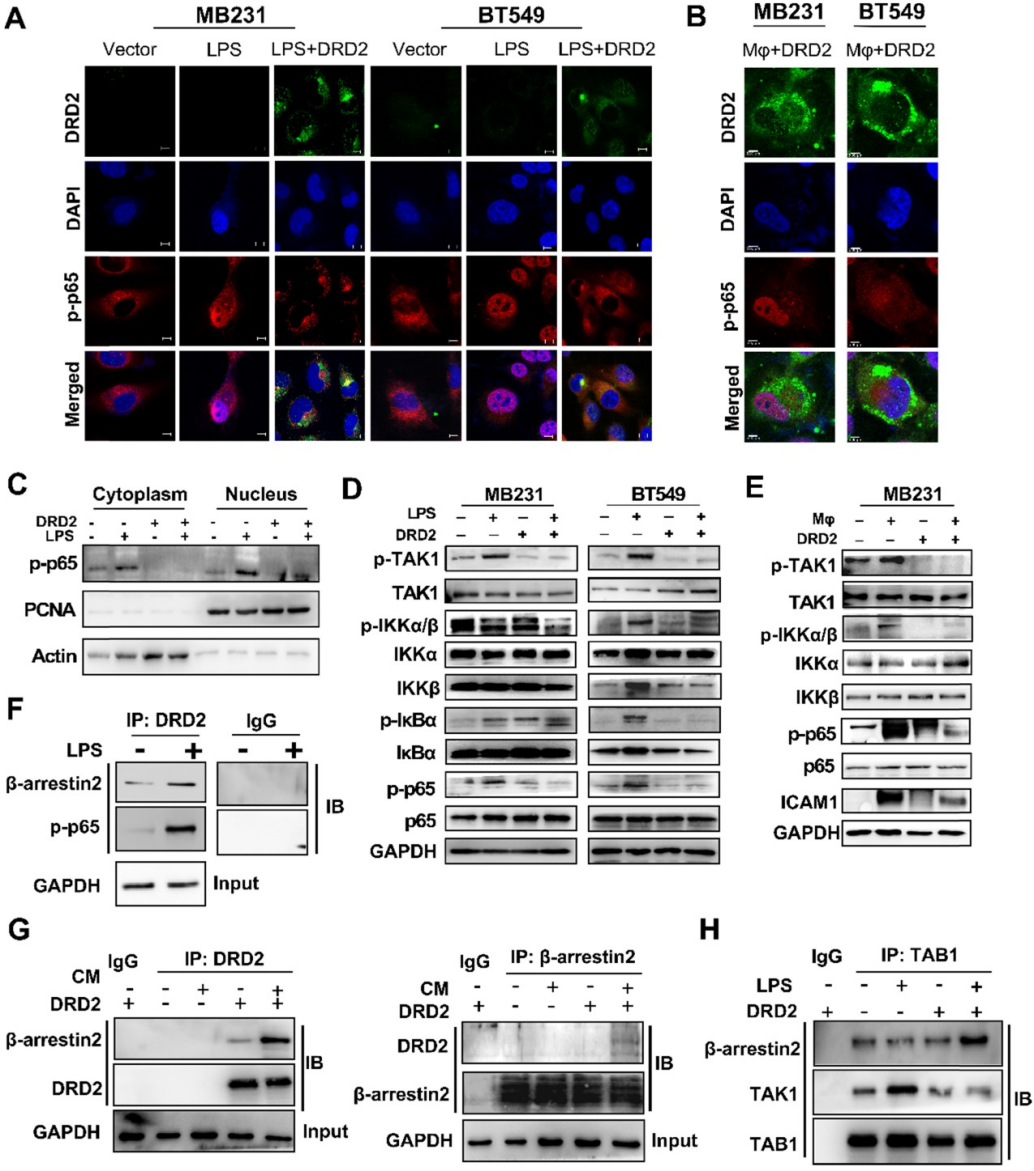
** DRD2 restricts NF-κB signaling activation by interrupting phosphorylation of TAK1.** (**A** and **B**) Representative IF staining of DRD2 (green) and p-p65 (red) in BrCa cells treated by LPS (A) or THP1-derived Mφ (B). And BrCa cells were seeded in glass coverslips for 24h before LPS (5 μg/ml, serum-free, 24 h) or Mφ treatment. Images were taken by confocal microscopy. Vector-transfected BrCa cells were used as the control. Nuclei were stained with DAPI. Bars, 10 μm in (A); 5 μm in (B). (**C**) WB was applied to detect cytoplasmic and nuclear expression of p-p65. PCNA and Actin were used to prove the protein integrity of nucleus and cytoplasm respectively. (**D** and **E**) WB was used to analyze the activation status of NF-κB signaling and its upstream regulator TAK1 after being treated by LPS (5 μg/ml, serum-free, 24 h) (D) and THP1-derived Mφ (3 d) (E). (**F** and **G**) IB was used to confirm the binding of proteins obtained by Co-IP in MDA-MB231. Cells were treated with LPS (5 μg/ml, serum-free, 24 h) or CM (3 d) before Co-IP. The binding of DRD2, β-arrestin2 and p-p65 were analyzed by IB in DRD2-expressed MDA-MB231 with or without LPS treatment (F). The binding of DRD2 and β-arrestin2 were analyzed by IB in in Vector- and DRD2-expressed MDA-MB231 with or without CM treatment (G). (**H**) IB was sued to determine the binding of TAB1, TAK1, and β-arrestin2 in Vector- and DRD2-expressed MDA-MB231. Co-IP was used to obtain possible binding proteins of TAB1 in samples with or without LPS treatment. IgG was used as negative control, and the input protein was used as positive control. And GAPDH was used for protein integrity. CM, Conditioned Medium.

The activation of NF-κB signaling is essential to trigger inflammasome assembly and subsequently pyroptosis 17. And experiments were applied to explore DRD2 effects on NF-κB activation. As TME is complex and contains series cytokines as well as chemokines, LPS, a classical NF-κB pathway stimulus, was used to investigate molecular mechanism as well. IF staining indicated DRD2 almost blocked the nuclear translocation of p-p65 **(Figure 5A)**, but this inhibition was counteracted by Mφ **(Figure 5B)**. Extracts of nucleus and cytoplasm also indicated DRD2 inhibited nuclear translocation of p-p65 **(Figure 5C)**. As shown in WB results, DRD2 suppressed the phosphorylation of IKKα/β, IκBα and p65 with LPS stimulation **(Figure 5D)**. And ectopic DRD2 expression also prominently inhibited Mφ-induced phosphorylation of p65 and the upstream activation of IKKα/β **(Figure 5E)**. WB results showed that DRD2 downregulated ICAM-1, the downstream target of NF-κB 18. However, this downregulation was counteracted by Mφ **(Figure 5E)**. The inhibition of the phosphorylation of IKKα/β suggested DRD2 negatively mediated the upstream of IKK complex. TAK1 plays a critical role in catalyzing IKKα and IKKβ 19, 20. The phosphorylation of TAK1 was significantly suppressed by the ectopic DRD2 expression **(Figure 5D-E)**. In general, DRD2 inhibits NF-κB signaling activation by interrupting its upstream kinase TAK1.

### DRD2 is triggered to internalization during the crosstalk with Mφ

DRD2 is generally located in the cell membrane without ligand activation, and Mφ could induce translocation of DRD2 during crosstalk **(Figure S4A)**. Subcellular trackers indicated that DRD2 could be induced to translocate to lysosomes by Mφ or LPS **(Figure S4B)**. And the protein expression level was downregulated by conditioned medium (CM) according to the WB results **(Figure S4C)**. Treatment with CHX confirmed the degradation of DRD2 protein by CM from the co-culture system **(Figure S4D)**. And THP1-derived Mφ exhibited subcellular translocation of DRD2 in the absence or presence of BrCa cells **(Figure S4E)**. The results described above suggested DRD2 could internalize or be stimulated endocytosis by non-selective ligands including hydrosoluble element from TAMs and LPS.

### DRD2-activated β-arrestin2 impairs the binding of TAK1 and TAB1

As a typical G protein-coupled receptor (GPCR), the internalization of DRD2 induces the plasma membrane recruitment of its adaptor β-arrestin2 and increases its affinity to bind to β-arrestin2 21-23. The protein-protein binding of DRD2 and β-arrestin2 was stimulated by LPS and CM **(Figure 5F-G)**. Meanwhile, DRD2 appeared to bind to p-p65 in the cytoplasm with LPS treatment as observed IF staining **(Figure 5A)**, and the binding was further confirmed by Co-IP and IB **(Figure 5F)**. It was reported that the activation of the β-arrestin2 signal disrupts TAK1-TAB1 binding in astrocytes, and this binding is essential for the activation of TAK1 24. This study also confirmed that the internalized DRD2 could promote the binding of TAB1 to β-arrestin2, and impair the binding of TAB1 to TAK1 **(Figure 5H)** Above all, DRD2-activated β-arrestin2 antagonizes the phosphorylation of TAK1 by competitively binding to TAB1.

### DRD2 inhibits NF-κB pathway activation and tumorigenesis by downregulating DDX5 and eEF1A2

**Figure 6:**
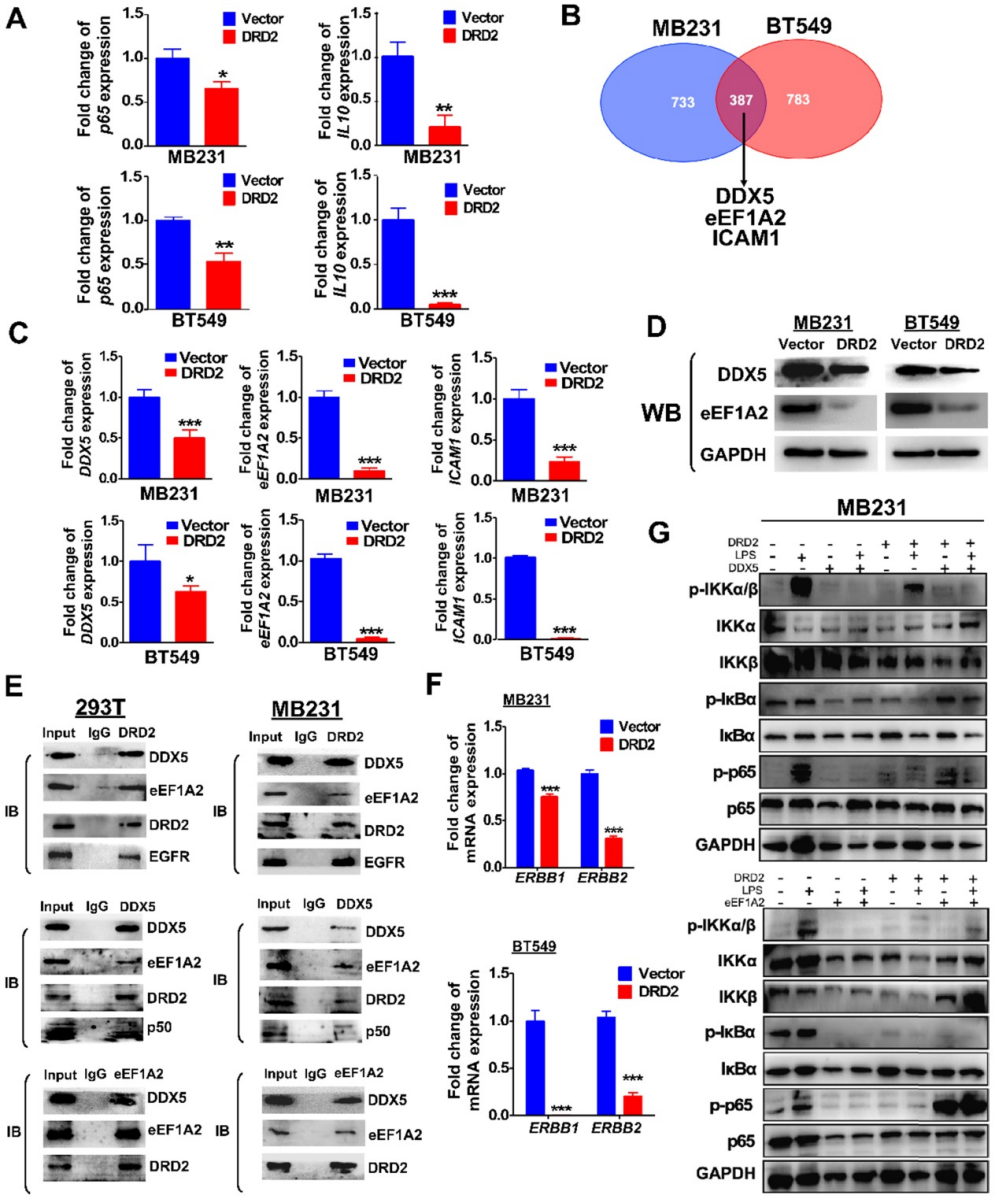
** DRD2 antagonizes NF-κB signaling through interacting and downregulating DDX5 and eEF1A2.** (**A**) qRT-PCR was used to detect mRNA expression of p65 and IL-10 in Vector- and DRD2- transfected BrCa cells. (**B**) Venn diagram showed potential binding proteins of DRD2 identified by mass spectrum analysis. And mass spectrum was performed in Co-IP isolated proteins in both MDA-MB231 and BT549. (**C**) mRNA expression of *DDX5*, *eEF1A2*, and *ICAM-1* were analyzed by qRT-PCR in Vector- and DRD2- transfected BrCa cells. (**D**) Protein expression of DDX5 and eEF1A2 were determined by WB in Vector- and DRD2- transfected BrCa cells. The expression was analyzed in both MDA-MB231 and BT549. (**E**) The binding of DRD2, DDX5 and eEF1A2 was examined by Co-IP and IB in 293T and MDA-MB231. (**F**) *ERBB1* (EGFR) and *ERBB2* (HER2) mRNA expression were determined by qRT-PCR in Vector- and DRD2- transfected BrCa cells. (**G**) WB was used to detect the effect of DDX5 and eEF1A2 on NF-κB activation was analyzed by WB in MDA-MB231 by stimulation of LPS. GAPDH was used for protein integrity. Data are presented as mean ± SD from biological replicates. P-value was calculated using two-tailed Student's t test. *, *p* < 0.05 **, *p* < 0.01; ***, *p* < 0.001.

Ectopic DRD2 expression significantly suppressed the mRNA expression of *p65* and NF-κB target genes *IL-10* **(Figure 6A)**, suggesting that DRD2 is a negative regulator of NF-κB pathway in the absence of ligands activation. Besides binding to activate β-arrestin2, DRD2 might suppress NF-κB signaling in other manners. To investigate the mechanisms, possible binding proteins were isolated by Co-IP, and MS analysis was applied to identify proteins. Among all the binding proteins in both MDA-MB231 and BT549 cells, *DDX5*, *eEF1A2* and *ICAM-1* were all confirmed to be downregulated by the ectopic DRD2 expression **(Figure 6B-C)**. DDX5 and eEF1A2 are two oncogenes in several cancer types according to previous researches 25-27. The downregulated protein level of DDX5 and eEF1A2 was also confirmed by WB **(Figure 6D)**. The binding of DRD2, DDX5 and eEF1A2 was confirmed by Co-IP and IB assay in both 293T and MDA-MB231 **(Figure 6E)**, indicating that these three proteins formed a complex. According to previous studies, DDX5 could bind to p50 and aid the release of p50 from IκB 28. This study also revealed the binding between DDX5 and p50 in both 293T and MDA-MB231 **(Figure 6E)**. Furthermore, DRD2 has been reported to bind to EGFR in the nervous system 29. In BrCa cells, the binding of DRD2 and EGFR was also confirmed in both 293T and MDA-MB231 **(Figure 6E)**. And DRD2 expression downregulated *ERBB1* (EGFR) and *ERBB2* (HER2) expression **(Figure 6F)**, and they are also target genes of NF-κB 30, 31. And in BrCa cells with ectopic DRD2 expression, DDX5 was found to promote the phosphorylation of p-IκBα and increase the protein levels of phosphorylation p65 **(Figure 6G)**. eEF1A2 was proved to strongly increase p-p65 directly without affecting IKKα/β or IκBα, and eEF1A2 even upregulated protein levels of p-p65 in the absence of LPS in DRD2-expressing MDA-MB231 **(Figure 6G)**. Results above showed the dramatical effects of DDX5 and eEF1A2 on promoting NF-κB signaling activation were suppressed by the ectopic DRD2 expression.

Furthermore, the ectopic expression of DDX5 and eEF1A2 **(Figure S5A-B)** also weakened the inhibitory effects of DRD2 on tumor proliferation as assessed by CCK8 **(Figure S5C-D)** and Transwell® assay **(Figure S5E)**. The results described above further confirmed that DRD2 suppresses NF-κB signaling activation and exerts tumor suppressive role through downregulating of DDX5 and eEF1A2 in BrCa cells.

## Discussion

TSGs are critical in the mediation of tumorigenesis 3, 4. Thus, identifying new TSGs and addressing unknown molecular events in BrCa is essential to characterizing carcinogenesis mechanisms, establishing novel prognostic biomarkers and even developing efficient therapeutic targets. TSGs are characterized by various features including downregulated expression and frequent DNA promoter methylation in tumors. In this study, to identify and characterize a new TSG impacting BrCa tumorigenesis, BrCa tissues as well as normal breast tissues were employed to RNA-seq screening. DRD2 was determined for further exploration by analyzing RNA-seq profile and online databases, which might benefit comprehensive treatment of BrCa patients. DRD2 is a dominated member of D2-like receptors and plays a significant role in memory and learning in nervous system 7. And several specific DRD2 agonists like bromocriptine have already used clinically in nervous system disorder 32, 33, and molecular mechanism underlying DRD2 anti-tumor effects might facilitate to breast cancer treatment by using selective DRD2 agonists. There are two families of dopamine receptors, D1-like and D2-like family. Besides DRD2, DRD3 and DRD4 also belong to D2-like family. The two classes of receptors induce distinct biochemical reactions. D1-like receptors increase intracellular level of cAMP, whereas D2-like receptor decreases cAMP levels 34, 35. Through analyzing expression and promoter methylation status, DRD2 was considered as a potential TSG for further investigation instead of DRD3 or DRD4. In BrCa, DRD2 was transcriptional downregulated by promoter methylation. RT-PCR detected unmethylated alleles in three breast cancer cell lines , suggesting that other transcription regulatory mechanisms, such as histone modification or transcriptional repression, also contribute to gene silencing 36. This study also identified the tumor-suppressive effect of DRD2 *in vitro* and *in vivo*.

In the present study, DRD2 could facilitate M1 phenotype Mφ. DRD2 has been confirmed to be involved in immune regulation 10. TAMs are the most abundant cell type in TME, which are plasticity and can be repolarized by various stimuli 37. Previous studies have manifested anti-inflammatory role of DRD2 in nervous system 38. In acute pancreatitis, myeloid-specific D2 signalling inhibited M1 macrophages through NADPH oxidase-mediated mitigating NF-κB 39. But DRD2 was also found to be essential in DA-reprogrammed M1 Mφ in glioma, which could be reversed by the DRD2 antagonist or deletion 40. Moreover, the inhibition of NF-κB signaling in TAMs would switch polarization of Mφ to M1 phenotype through mediating IKKβ 41. DRD2 might trigger different polarization of macrophage in inflammation disease and in carcinoma, even in different cell types. In this research, although TNF-α and IFN-γ are critical to induce M1 Mφ, this study showed no significant increase of TNF-α and IFN-γ in DRD2-expressing BrCa cells during crosstalk with Mφ. Instead, this study has revealed that DRD2 significantly suppressed both IL-6 and IL-10 expression in BrCa. IL-6 is abundant in TME and has been shown to induce differentiation of M2 macrophages 42. IL-10 is also significant in inducing M2 polarization and can enhance M2 phenotype attributable to another stimulus 37, 43. Thus, the dramatically downregulation of IL-6 and IL-10 might trend to polarize Mφ to the M1-like phenotype. DRD2-transfected BrCa cells also generated NLRP3 inflammasome and activated caspase-1 during the co-cultivation, which further produced mature IL-1β and IL-18. Both IL-1β and IL-18 are involved in anti-tumor immunity and are capable of educating Mφ to M1 phenotype 44, 45. The assembly of NLRP3 inflammasome and production of mature IL-1β and IL-18 further explain how DRD2-expressing BrCa cells educated Mφ to M1 polarization during the crosstalk.

DRD2 also induced apoptosis as well as necroptosis. DRD2 further triggered assembly of NLRP3 inflammasome and pyroptosis during crosstalk with Mφ. GSDMD have redefined pyroptosis as gasdermin-mediated programmed necrosis 46. This study manifested that GSDME, instead of GSDMD, was the executioner of DRD2-triggering pyroptosis in BrCa cells. GSDME has been also reported to induce anti-tumor immunity with killer lymphocytes and TAMs 47, which might also facilitate M1 phenotype Mφ during crosstalk. DRD2 was previously reported to inhibit inflammasome assembly in nervous system 38, whereas this study indicated that DRD2-educated Mφ triggered GSDME-executed pyroptosis in a DRD2-dependent manner during co-cultivation. Ectopic expression of DRD2 also activated caspase-8 and pMLKL. Caspase-8 was revealed to be the molecular switch for apoptosis, necroptosis and pyroptosis 48, 49, and activated pMLKL was also reported to triggered NLRP3 inflammasome in a cell-intrinsic manner 50. Results above suggested that DRD2 is essential to trigger PCD. More studies are necessary to explore how DRD2 links different PCD and changes processes of PCD. And the molecular mechanism of DRD2-induced pyroptosis provide new theoretical foundation for further researches.

NF-κB signaling pathway is essential to trigger pyroptosis 17. Previous studies have suggested that DRD2 is a crucial anti-inflammatory factor in nervous system diseases by suppressing of NF-κB activation 51, 52. In this study, ectopic expression of DRD2 antagonized the activation of NF-κB signaling, which was counteracted by Mφ. And DRD2-educated Mφ mediated the induction of inflammasome and pyroptosis in a DRD2-dependent manner. DRD2 belongs to (GPCRs), and activated GPCRs is involved in regulating the flow of second messengers like cAMP 53, 54. Previous studies indicated DRD2 could decrease cellular level of cAMP 55, 56, while cAMP/PKA regulates transcriptional activities of NF-κB 57. In non-small cell lung cancer progression (NSCLC), DRD2 was reported to block NF-κB signaling pathway by regulating cAMP/PKA/p65 axis 55. Besides cAMP, GPCRs also initiate G protein-independent signals by β-arrestins like β-arrestin2 54. β-arrestin2 is critical adaptor of DRD2, which also regulates internalization of GPCRs and inflammatory responses 58, 59. In the present study, DRD2 was induced endocytosis by Mφ during the crosstalk. The internalized DRD2 bind to β-arrestin2, and activated β-arrestin2 was confirmed to bind to TAB1 competitively and further inhibit phosphorylation of TAK1, which was consistent with previous studies 20, 21, 24, 60. TAK1, also known as Mitogen-Activated Protein Kinase Kinase Kinase 7, is a member of the MAPKKK family, and mediates the phosphorylation of IKKα and IKKβ 19, 20**.** Meanwhile, DRD2 was found to bind to p-p65, and this binding also explains the prevention of nuclear translocation of p-p65. And DRD2-suppressed ICAM-1 was upregulated by Mφ. ICAM-1 is a cell surface glycoprotein. ICAM-1 was reported to regulate anti-tumor immunity *in vivo* 61, but it was also found to promote metastatic ability of breast cancer cells *in vitro* 62. Interestingly, ICAM-1 is also a NF-κB targeted gene 18. Results above demonstrated the critical role of DRD2 in restricting NF-κB signaling activation. NF-κB signaling plays diverse and complex role in tumors 63, and DRD2 might mediate this signaling to exert anti-tumor effects. And DRD2 could be activated by non-selective ligands even by BrCa-associated Mφ which makes DRD2 a promising therapeutic target in BrCa.

In breast cancer, DRD2 was found to exert anti-tumor effects when it locates in cellular membrane and in cell. When DRD2 located in cellular membrane, it exerted anti-tumor effects through downregulating DDX5 and eEF1A2. When DRD2 was activated by agonists, it exerts anti-tumor effects through ROCK-mediated cofilin inactivation or EGFR/AKT/MMP-13 pathway 9, 11. And the internalized DRD2 was found to bind to β-arrestin2. In nervous system, DRD2 expression at the cell membrane is essential to active the dopamine signaling pathway. DRD2-mediated signaling is mainly regulated by endocytosis 64, and β-arrestin2 is essential to target DRD2 for internalization 65. Internalization of DRD2 is finely regulated to recycle back to plasma member or translocate to lysosome for degradation 66, while impaired internalization was considered to associate with schizophrenia 67.

Other possible binding proteins of DRD2 were isolated and identified by of Co-IP and MS. DDX5, eEF1A2 and ICAM-1 were all found to bind to DRD2 and to be downregulated by DRD2. And these three proteins were all shown to be in close relationship with the NF-κB signaling pathway 28, 68. DDX5, a conserved protein belonging to DEAD box family of RNA helicases, is involved in regulating various factors, including c-Myc, AKT, and NF-κB 26, 28. eEF1A2 was a eukaryotic elongation factor, and was reported as a potential oncogene in ovarian cancer 69. And eEF1A2 was also found to promote migration and invasion in a largely PI3K- and Akt- dependent manner 70**.** In this study, DRD2 was confirmed to suppress tumorigenesis if breast cancer through interacting with DDX5 and eEF1A2. Furthermore, this study demonstrated that eEF1A2 upregulated protein levels of phosphorylation of p65 without affecting IKK complex or IκBα, and even strongly upregulated protein level of phosphorylation of p65 in the absence of LPS. And DDX5 could promote phosphorylation of IκBα. Generally, the interaction of DRD2 and its binding proteins finally inhibits NF-κB signaling in the upstream of NF-κB signaling and phosphorylation of p65. On the one hand, DRD2 antagonizes NF-κB signaling activation; on the other hand, DRD2 edits Mφ to M1 phenotype and promotes NF-κB-initiated pyroptosis during the crosstalk. DRD2 might restrict NF-κB signaling to regulate anti-tumor effects.

Diagnosis in advanced tumor stage and recurrence are two major causes of poor survival in cancer patients, and the treatment for recurrence and metastasis still lacks advances 1, 2. The overexpression of EGFR indicates a poor prognosis in BrCa. In previous studies, DRD2 negatively regulated EGFR signaling 11, 29. EGFR is a significant therapeutic target, whereas clinical treatment of EGFR has shown poor results because of chemoresistance 71. In this study, DRD2 was more common in HER2-negtive patients, and DRD2 also promoted survival times of HER2-positive patients. DRD2 was found to be a negative regulator of EGFR and HER2, and DRD2 expression might facilitate treatment of HER2-positive BrCa patients. Further researches are necessary to state the details of the molecular mechanism. PTX is the first line chemotherapy agent used in BrCa patients, whereas acquired resistance has been reported recently 72. DRD2 expression promoted drug sensitivity to PTX and caused increased cell death, which indicated that DRD2 also functioned as a biomarker for precise PTX treatment. For the potential therapeutic role of DRD2, more researches are needed like applying Bromocriptine, a proved DRD2 agonist used for Parkinson's disease. And combination therapy of Aza and Bromocriptine might promote outcome of BrCa patients with silenced or low expression of DRD2. The present findings suggest that DRD2 is a potential biomarker for predicting prognosis and precise treatment.

In summary, this study novelly identified a TSG, DRD2, improves survival and PTX treatment response of BrCa patients. DRD2 induces apoptosis as well as necroptosis, and further triggers pyroptosis during reprogramming Mφ to M1. DRD2 restricts NF-κB signaling activation by binding to β-arrestin2, and downregulating DDX5 as well as eEF1A2. These findings suggest that DRD2 is a potential biomarker for predicting prognosis, and DRD2 is a promising therapeutic target in BrCa.

## Materials and method

### Tissue specimens

The BrCa tissues and normal breast tissues were all obtained from the First Affiliated Hospital of Chongqing Medical University (CQMU) and RNA was extracted for RNA-seq screening. All samples were reviewed and subjected to histological as reported previously 73, 74.

### Immunohistochemistry (IHC)

Samples from both patients and mice were studied following a previously published protocol 75. And antibodies used were listed on **Table 3**. After the final staining, the samples were scanned using an IHC scanner (PANNNORAMIC MIDI, Budapest, Hungary) and processed by CaseViewer (v2.2.0.85100, Budapest, Hungary). To detect Mφ markers, serial sections from human samples were used to perform IHC.

### Reverse transcription (RT)-PCR and real-time PCR (qRT-PCR)

Genomic RNA and total DNA were isolated from cell lines and tissues using TRI Reagent® (Molecular Research Center, Cincinnati, OH, USA) and DNAzol® reagent (Invitrogen, Rockville, MD), respectively, according to the manufacturer's instructions. The concentration of the samples was determined by spectrophotometry using a NanoDrop^TM^ 2000 (Thermo scientific). Reverse transcription of RNA was performed using GoScript^TM^ reverse transcriptase (Promega, Madison, WI) and reaction conditions were the same as previously reported 4. Semi-quantitative PCR (RT-PCR) was conducted using the Go-Taq system (Promega, Madison, WI, 487 USA) under the conditions detailed in a previous study 74. Real-time PCR (qRT-PCR) was performed using SYBR (Promega) according to the instrument manual (HT7500 System; Applied Biosystems, Foster, USA). Relative expression was calculated using the 2^-△Ct^ method. *GAPDH* were amplified as controls for RNA integrity. The sequences of primers and reaction systems are listed in **Table 4**.

### Cell culture and reagents

BrCa cell lines (MDA-MB231, BT549, YCCB1, 4T1, etc.), immortalized human mammary epithelial cell lines (MCF-10A, HMEC) and HEK293T were obtained from the American Type Culture Collection (ATCC, Manassas, VA, USA) or collaborators and cultured in PRMI 1640 (Gibco) or DEME medium supplemented with 10% fetal bovine serum (FBS, Gibco) and 1% penicillin-streptomycin (Gibco) according to standard protocols. LPS (5 μg/ml, Beyotime) was used to activate the NF-κB signal pathway in serum-free PRIM 1640 for 24 h in BrCa cell lines. Quinpirole (2μM, Cas #: 85798-08-9, Sigma-Aldrich) was applied to selectively activated DRD2. si-RNA used for knocking down DRD2 was purchased from Origene according to its Application Guide. CHX (50 μg/ml, Sigma-Aldrich) weas used to treated BrCa cells.

### Methylation analysis

MDA-MB231 and BT549 were treated with Aza (Sigma-Aldrich) 76. Cells were collected for RNA and DNA analyses. Methylation-specific PCR (MSP) were used to detect promoter methylation. AmpliTaq-Gold DNA polymerase was applied to serve the goal of amplifying target genes. And 2% agar gel was used for electrophoresis, and imaging was photographed. Primer used for MSP were listed **Table 4**.

### Construction of stable cell lines

The full length DRD2 gene with a Flag tag was inserted into a pcDNA3.1(+) framework plasmid, and the plasmid was recombined as in previous work 73. Lipofectamine3000 (Invitrogen, CA) and Opti-MEM (Invitrogen, CA) were used for transfection following the manufacturers' instructions. MDA-MB231 and BT549 were transfected with DRD2 plasmids and filtrated with G418 to establish stably overexpressing-DRD2 cell lines. The pcDNA3.1-empty plasmid was transfected into generated control cell lines. RT-PCR and Western Blot (WB) were performed to confirm ectopic expression of DRD2.

### Western Blot (WB)

WB was performed as previously described work 77. Primary antibodies used in WB were all listed on **Table 3**. And Restore™ Western Blot Stripping Buffer (#21059, Thermo Fisher) was used to strip protein mixture on the PVDF membranes according to manufacturer's protocol. The chemiluminescence kit (Amersham Pharmacia Biotech, Piscataway, NJ) was used to visualize protein bands in a Gel Imager System (FX5, Vilber Lourmat).

### Cell proliferation assay

The growth rate of cells was measured at 0, 24, 48, and 72 h with the Cell Counting Kit-8 (CCK-8; Beyotime, Shanghai, China). The monolayer colony formation assay was used to test cellular anchorage-dependent growth *in vitro*. Ectopic DRD2-expressing cells or vector-transfected cells were plated in a six-well plate (MDA-MB231, 800 cells/well; BT549, 1200 cells/well). Surviving colonies (≥50 cells per colony) were visualized with gentian violet staining and counted. The soft agar assay was used to investigate cellular anchorage-independent growth *in vitro* and performed as previously described in published research 73. Cell colonies were photographed and counted under a 10× Leica microscope after 2-3 weeks of incubation. And paclitaxel (PTX, MACKLIN, P875571) was applied at centration of 20μg/ML for 24 h and DMSO was used as control.

### Cell cycle and apoptosis analyses

Flow cytometry (FC) analysis was applied to assess the cell cycle. Cells were cultured (10×10^5^) in six-well plates for 48 h, and were subsequently collected, fixed and analyzed using CELL Quest software (BD Biosciences, San Jose, CA) as described previously 74. An acridine orange/ethidium bromide kit (AO/EB) (LEAGENE) was used to analyze apoptosis following the instructions. Stained cells were visualized under a fluorescence microscope (Leica CTR4000B, Leica Microsystem, USA). The apoptosis rate (%) = (apoptotic cells/total cells) × 100%.

### Mobility assays

Both a wound-healing assay and Transwell® assays were performed to investigate the mobility of the cancer cells. MDA-MB231 and BT549 cells with stable DRD2 expression were seeded into six-well plates, and the vector-transfected cells were used as controls. When the cells were confluent, a sterilized pipette tip was used to cause cellular wounds artificially. After washing three times with PBS, cells continued to be cultured with RPMI medium without FBS. Transwell® assays with or without coated Matrigel (BD, Biosciences Discovery Labware) were carried out as reported previously 74. All images were obtained using a microscope (Olympus, Tokyo, Japan) and then measured or counted.

### Subcutaneous tumor model in BALB/c mice

BALB/c mice (aged of 6-8 weeks) were purchased and reared according to ethical guidelines by the Experimental Animal Center of Chongqing Medical University (CQMU), China. A murine breast cancer cell line 4T1 stably expressing DRD2 and vector-empty cells (1×10^6^ cells in 0.1ml PBS per mouse) were injected subcutaneously into the lower backs of BALB/c mice (8 mice per group). The longest and shortest diameters of tumors were measured using a Vernier caliper every 3 d for 12 d. Tumor volume (mm^3^) was calculated as follows: volume = length × width^2^ × 0.5. The mice were killed before the volume of their tumor reached 1 cm^3^. The tumors were extracted, photographed and conserved as paraffin samples.

### Co-cultivation of BrCa cells and macrophages

The co-cultivation of BrCa cells and Mφ was constructed using a non-contact co-culture Transwell® system (#3450, Corning, USA). Human macrophages were derived from monocytic THP-1 (Cell Bank of Typical Culture Preservation Commission, Chinese Academy of Sciences). To generate macrophages, 1×10^6^ THP-1 cells were seeded in the bottom chamber of a Cell Culture Insert with PRIM medium containing 10 ng/ml 12-myristate 13-acetate (PMA, Sigma) for 24 h 78. Then the medium was replaced by PRIM medium. BrCa cells with or without DRD2 overexpression were seeded into the upper chamber and co-cultured with Mφ for 72 h before harvest. And the CM used to treat BrCa cells and detect cytokines was harvested after 3 d of co-cultivation. The primary Mφ derived from THP-1 (M0) were used as control. To generate M1 polarized Mφ, THP1-derived Mφ were treated with LPS (200 ng/ml, Beyotime) for 3 d. After washing with PBS, the cells were incubated for 24 h. All the CM was filtered before use.

### Immunofluorescence (IF)

Cells were seeded on glass coverslips and cultured for multiple time based on the objective of the experiment. For most samples, the cultivation lasted for 48 h. And for co-cultured samples, the cultivation lasted for 3 d. They were double stained according to a previously published protocol 74, and DAPI was used as a nuclear counterstain. The primary antibodies and second antibodies used were all listed on **Table 3**. For subcellular location detection, ER-Tracker Red (C1041, Beyotime) was applied following by manufacturer's protocols. Images of the samples were taken using a confocal laser scanning microscope (Leica Microsystems CMS GmbH Am Friedensplatz 3, 68165 Mannheim Germany). And imaging's morphology of BrCa cells was photographed in bright field using a confocal laser scanning microscope mentioned above.

### Cytokine detection

Culture medium of BrCa cells was tested for secreted cytokines with or without co-cultivation with THP1-derived Mφ. For collection of culture medium, groups of cells were washed with PBS for three times and cultivated with serum-free RPMI before harvesting. Cells co-cultured with Mφ were cultured alone for another 24 h after terminating co-cultivation; all the medium was centrifuged for 20 min at 1000 ×g at 4°C, and the supernatant used. The methods used for the antibody array (#GSH-TH-1, RayBiotech, Norcross, GA) completely followed the manufacturer's instructions. And was detected fluorescence by RayBiotech (RayBiotech, Inc., Guangzhou) and the Analysis Tool specific for this array is catalog number: GSH-TH-1-SW.

### TUNEL assay

The TUNEL (terminal deoxynucleotidyl transferase) assay was applied to examine apoptosis in samples from mice. A TUNEL detection kit (Beyotime) was used and results were observed using a confocal laser scanning microscope (Leica Microsystems CMS GmbH Am Friedensplatz 3, 68165 Mannheim Germany). The experiment followed the manufacturer's protocol.

### Nuclear and Cytoplasmic Extraction

Separation and preparation of cytoplasmic and nuclear extracts were obtained used NE-PER Nuclear and Cytoplasmic Extraction Reagents (78833, Thermo Scientific) following manufacturer's protocol. All samples and extracts were kept on ice, and extracts were stored at -80℃. PCNA (sc-56, Santa Cruz) and Actin (sc-8432, Santa Cruz) were used for nuclear and cytoplasmic protein integrity respectively.

### Co-immunoprecipitation (Co-IP), and mass spectrometry (MS) analysis

Co-IP was performed to confirm protein-protein binding. MilliporeSigma™ PureProteome™ Protein A/G Mix Magnetic Bead System (#LSKAGAG10, Fisher Scientific, USA) and detailed protocol was published on the former report 73. The antibodies used to incubate cell lysate was listed on **Table 3**. The Co-IP complexes were analyzed by SDS-PAGE and IB (Immunoblot). And anti-mouse IgG (#A25012, Abbkine) was used as second antibodies for Co-IP complexes detection and the Co-IP complexes. And the binding proteins of DRD2 were isolated by Co-IP and identified by mass spectrometry (MS) analysis applying Triple tof5600 and the results were processed by ProteinPilot (version 5.01).

### Bioinformation and statistical analysis

Expression and promoter methylation of DRD2 in BrCa were analyzed on TCGA online. And another online database, MethHC, was also applied to analyze the expression and methylation status of DRD2. Kaplan-Meier plots were accessed online. Characteristics of breast cancer patients, methylation and expression status of DRD2 were all obtained from The Cancer Genome Atlas (TCGA). SPSS22 (version 22.0, IBM, SPSS, Chicago) and GraphPad (version 6.0, GraphPad Prism Software, USA) were used for the statistical analyses. The Student's t-test, chi-squared test, Spearman correlation test, and Fisher's exact test were used to evaluate the assay results, assess relationships and compare methylation status.

## Supplementary Material

Supplementary figures.

## Figures and Tables

**Table 1:** Relationship between clinicopathological features and DRD2 expression in breast cancer (TCGA)

Clinicopathological features	Number (n = 602)	Low expression	High expression	χ2	p Value
**Age**				-0.11	**0.007**
> 55	364	200(54.95%)	164(45.05%)		
≤ 55	238	104(43.70%)	134(56.30%)		
**ER**				-0.019	0.64
Negative	135	66(48.89%)	69(51.11%)		
Positive NULL = 2	465	238(51.18%)	227(48.82%)		
**PR**				-0.006	0.892
Negative	191	96(50.26%)	95(49.74%)		
Positive	407	207(50.86%)	200(49.14%)		
NULL = 4					
**HER2**				-0.161	**0.000084**
Negative	501	234(46.71%)	267(53.29%)		
Positive	93	64(68.82%)	29(31.18%)		
NULL = 8					
**Metastasis**				-0.022	0.584
M0	590	297(50.34%)	293(49.66%)		
M1	12	7(58.33%)	5(41.67%)		
NULL = 0					
**Stage (AJCC)**				-0.025	0.535
I - II	450	224(49.78%)	226(50.22%)		
III - IV	146	77(52.74%)	69(47.26%)		
NULL = 6					

Null: indeterminated / equivocal, ER: estrogen receptor, PR: progesterone receptor, HER2: Human epidermal growth factor receptor 2, AJCC: American Joint Committee on Cancer.

**Table 2:** Relationship between clinicopathological features and DRD2 promoter methylation in breast cancer (TCGA)

Clinicopathological features	Number (n = 243)	Low methylation level	High methylation level	χ2	p Value
**Age**				0.138	**0.032**
> 55	155	93(60.00%)	82(40.00%)		
≤55	88	66(75.00%)	22(25.00%)		
**ER**				0.092	0.155
Negative	54	40(74.07%)	14(25.93%)		
Positive	187	119(63.64%)	68(36.36%)		
NULL = 2					
**PR**				0.072	0.265
Negative					
Positive	84	59(70.24%)	25(29.76%)		
NULL = 2	157	99(63.06%)	58(36.94%)		
**HER2**				0.124	0.056
Negative					
Positive	191	130(68.06%)	61(31.94%)		
NULL = 5	47	25(53.19%)	22(46.81%)		
**Metastasis**				0.022	0.736
M0					
M1	236	154(65.25%)	82(34.75%)		
NULL = 0	7	5(71.43%)	2(26.57%)		
**Stage (AJCC)**				0.002	0.977
I - II	183	119(65.03%)	64(34.97%)		
III - IV	54	35(64.81%)	19(35.19%)		
NULL = 6					

Null: indeterminated / equivocal, ER: estrogen receptor, PR: progesterone receptor, HER2: Human epidermal growth factor receptor 2, AJCC: American Joint Committee on Cancer

**Table 3:** List of antibodies used in this study

Experiment	Name	LOT	Manufacturer
IHC	iNOS	ab15323	Abcam
	CD206	ab8918	Abcam
	CD68	ab955	Abcam
	CD163	ab182422	Abcam
	DRD2	SAB4301831	Sigma-Aldrich
	Ki67	ab16667	Abcam
	pMLKL	ab196436	Abcam
WB	Vimentin	sc-6260	Santa Cruz
	E-cadherin	sc-8426	Santa Cruz
	ZEB1	sc-515797	Santa Cruz
	DRD2	sc-5303	Santa Cruz
	iNOS	ab15323	Abcam
	CD206	ab8918	Abcam
	ICAM-1	sc-8439	Santa Cruz
	p-NFκB p65	sc-136548	Santa Cruz
	NF-κB p65	sc-8008	Santa Cruz
	EF-1 α1/2	sc-377439	Santa Cruz
	DDX5	sc-365164	Santa Cruz
	NF-κB Pathway Sampler Kit	3396	CST
	NLRP3	sc-134306	Santa Cruz
	DYKDDDDK Tag	14793	CST
	IL-1β	sc-12742	Santa Cruz
	IL-18	sc-133127	Santa Cruz
	NF-κB p50	sc-166588	Santa Cruz
	caspase-3	sc-271759	Santa Cruz
	caspase-1	YH050707C	Epitonics
	pMLKL	ab196436	Abcam
	GAPDH	sc-47724	Santa Cruz
	TAK1	5206	CST
	p-TAK1	4508	CST
	GSDME	ab215191	Abcam
	GSDMD	ab209845	Abcam
	TAB1	3226S	CST
IF	iNOS	ab15323	Abcam
	MH class II	sc-32247	Santa Cruz
	CD206	ab8918	Abcam
	p-NFκB p65	sc-136548	Santa Cruz
	DRD2	sc-5303	Santa Cruz
	DRD2	SAB4301831	Sigma-Aldrich
	Vimentin	sc-6260	Santa Cruz
	E-cadherin	sc-8426	Santa Cruz
	Anti-mouse IgG Alexa Flour® 594	ab150116	Abcam
	anti-mouse IgG Alexa Flour®488	ab150113	Abcam
	anti-rabbit IgG Alexa Flour®488	ab150077	Abcam
Co-IP	DYKDDDDK Tag	14793	CST
	TAB1	3226S	CST
	DDX5	sc-365164	Santa Cruz
	DRD2	sc-5303	Santa Cruz
	EF-1 α1/2	sc-377439	Santa Cruz
	β-arrestin2	sc-13140	Santa Cruz

IHC: Immunohistochemistry, WB: Western Blot, IF: Immunofluorescence, Co-IP: co-immunoprecipitation, CST: Cell Signaling Technology.

**Table 4:** List of PCR primers used in this study

PCR	Primer	Sequence (5'-3')	Product size (bp)
RT-PCR / qRT-PCR	*DRD2F*	ACTACCTGATCGTCAGCCTCG	118bp
	*DRD2R*	ATGTCACAGTGAATCCTGCTG	
	*GAPDHF*	GTGATGGGATTTCCATTGAT	206bp
	*GAPDHR*	GTGATGGGATTTCCATTGAT	
	*DDX5F*	CCTTGTCCTTGATGAAGCAG	151bp
	*DDX5R*	CAGGAAATCTTCAGCAAGCT	
	*eEF1A2F*	GGCCACCTCATCTACAAATG	170bp
	*eEF1A2R*	TCGAACTTCCAGAGGGAGAT	
	*ICAM1F*	GGTGTATGAACTGAGCAATGTG	102bp
	*ICAM1R*	CAGTACACGGTGAGGAAGGT	
	*IL10F*	GACTTTAAGGGTTACCTGGGTTG	112bp
	*IL10R*	TCACATGCGCCTTGATGTCTG	
	*IL1BF*	CTCCAGGGACAGGATATGGA	294bp
	*IL1BR*	TTCTGCTTGAGAG GTGCTGA	
	*EGFRF1*	GATGCTCTCCACGTTGCACAG	142bp
	*EGFRR1*	GGGGCACGATTGTCAAAGA	
	*HER2F*	ATGGAGCTGGCGGCCTTGTG	193bp
	*HER2R*	GGTAGGTGAGTTCCAGGTTT	
	*RELA-F*	GGGGCACGATTGTCAAAGA	119bp
	*RELA-R*	GGGGACTACGACCTGAATGC	
	*TNFAF*	ATGAGCACTGAAAGCATGATCCG	171bp
	*TNFAR*	GCCGATCACTCCAAAGTGCAG	
	*ARG1F*	TGAGCGCCAAGTCCAGAAC	114bp
	*ARG1R*	TCTCAAGCAGACCAGCCT	
	*mDRD2F*	ACTACCTGATAGTCAGCCTCG	120bp
	*mDRD2R*	AGATGTCACAGTGAATCCTGC	
	*mGAPDH*	CCAGCAAGGACACTGAGCAAG	77bp
	*mGAPDH*	ATGGAAATTGTGAGGGAGATGC	
MSP	*DRD2m1*	CGTTTAGGTCGGGGATCGTC	123bp
	*DRD2m2*	TCTACGACGCCCGAACGCG	
	*DRD2m3*	CGAATCGGTAGTTTACGCGC	140bp
	*DRD2m4*	CGACGAAACGAAACGAAACG	

Note. RT-PCR: Reverse Transcription-Polymerase Chain Reaction, qPCR: quantitative Polymerase Chain Reaction MSP: methylation-specific PCR

## Abbreviations

BrCa: breast cancer
CCK8: Cell Counting Kit-8
CHX: Cycloheximide
CM: conditioned medium
Co-IP: Co-immunoprecipitation
DA: dopamine
DDX5: DEAD-Box Helicase 5
DRD2: D2 Dopamine Receptor
DRD3: D3 Dopamine Receptor
DRD4: D4 Dopamine Receptor
eEF1A2: Eukaryotic Translation Elongation Factor 1 Alpha 2
EGF: Epidermal Growth Factor
EGFR: Epidermal Growth Factor Receptor
EMT: epithelial-mesenchymal transition
FC: flow cytometry
GPCR: G protein-coupled receptor
GSDME: Gasdermin E
HER2: Human Epidermal Growth Factor Receptor 2
ICAM-1: Intercellular Adhesion Molecule 1
IF: Immunofluorescence
IHC: immunohistochemistry
LPS: Lipopolysaccharide
Mφ: macrophages
MLKL: Mixed Lineage Kinase Domain Like Pseudokinase
MS: mass spectrum
NSCLC: non-small cell lung cancer progression
PCD: programed cell death
PTX: Paclitaxel
NLRP3: NLR Family Pyrin Domain Containing 3
qRT-PCR: real-time PCR
RNA-seq: RNA sequences
TAK1: Mitogen-Activated Protein Kinase Kinase Kinase 7
TAB1: TGF-Beta Activated Kinase 1 (MAP3K7) Binding Protein 1
TAMs: tumor associated macrophages
TCGA: The Cancer Genome Atlas
TME: tumor-associated microenvironment
TSG: tumor suppressor genes
TUNEL: TdT-mediated dUTP Nick-End Labeling
WB: western blot
