# Supplementary material for: Tumor suppressor DRD2 facilitates M1 macrophages and restricts NF-κB signaling to trigger pyroptosis in breast cancer
Source: Theranostics. 2021 Mar 5;11(11):5214–31. doi: 10.7150/thno.58322 (PMC8039962; doi:10.7150/thno.58322)
Supplement: Supplementary file 1 — Supplementary figures. [file thnov11p5214s1.pdf]

Supplementary Figures

Figure S1.

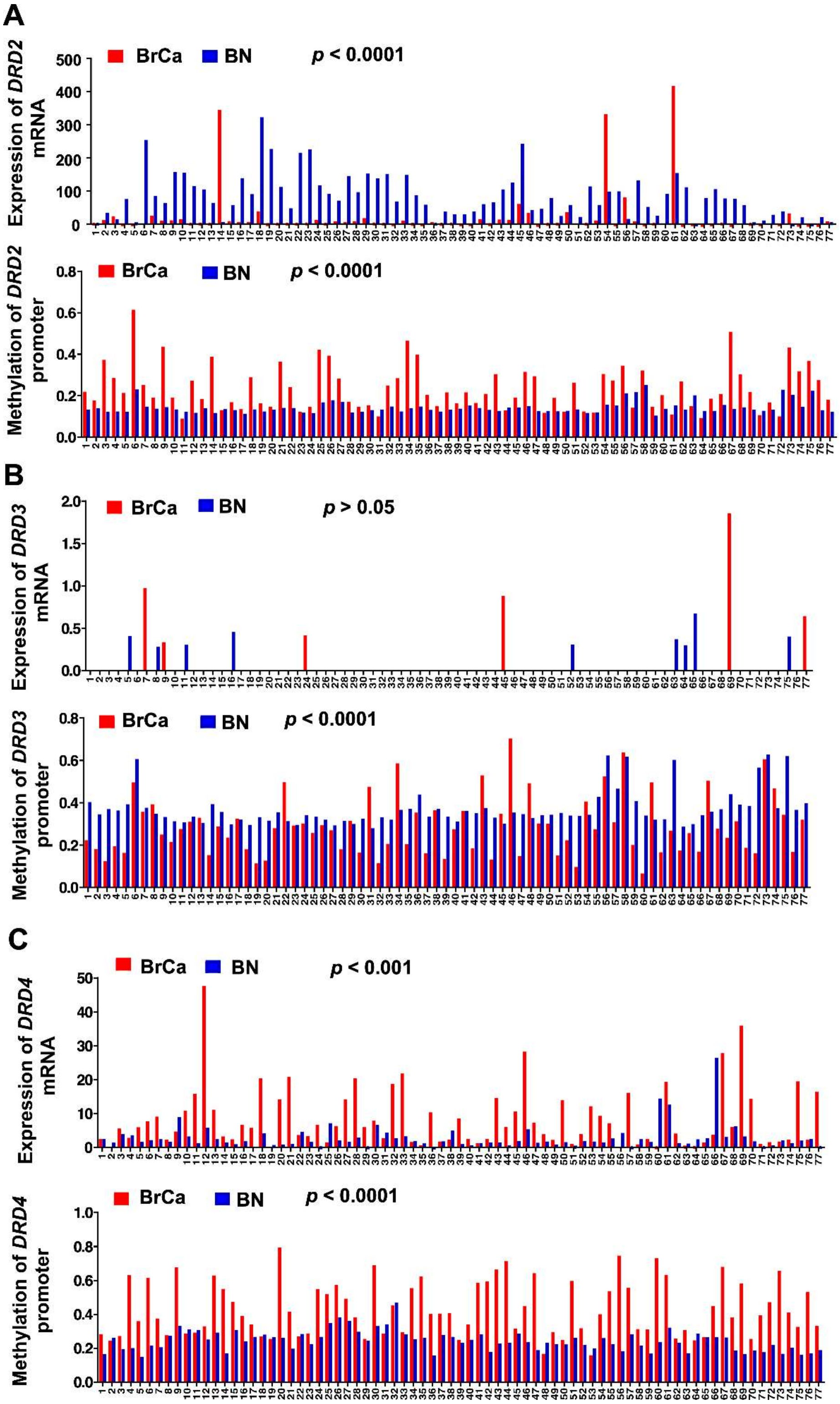

Figure S1. Expression and promoter methylation status of D2-like class receptors. (**A-C**). Expression and promoter methylation status of DRD2 (A), DRD3 (B), and DRD4 (C) were analyzed in BrCa and normal breast tissues by using online database MethHC. BrCa, breast cancer; BN, normal breast.

Figure S2.

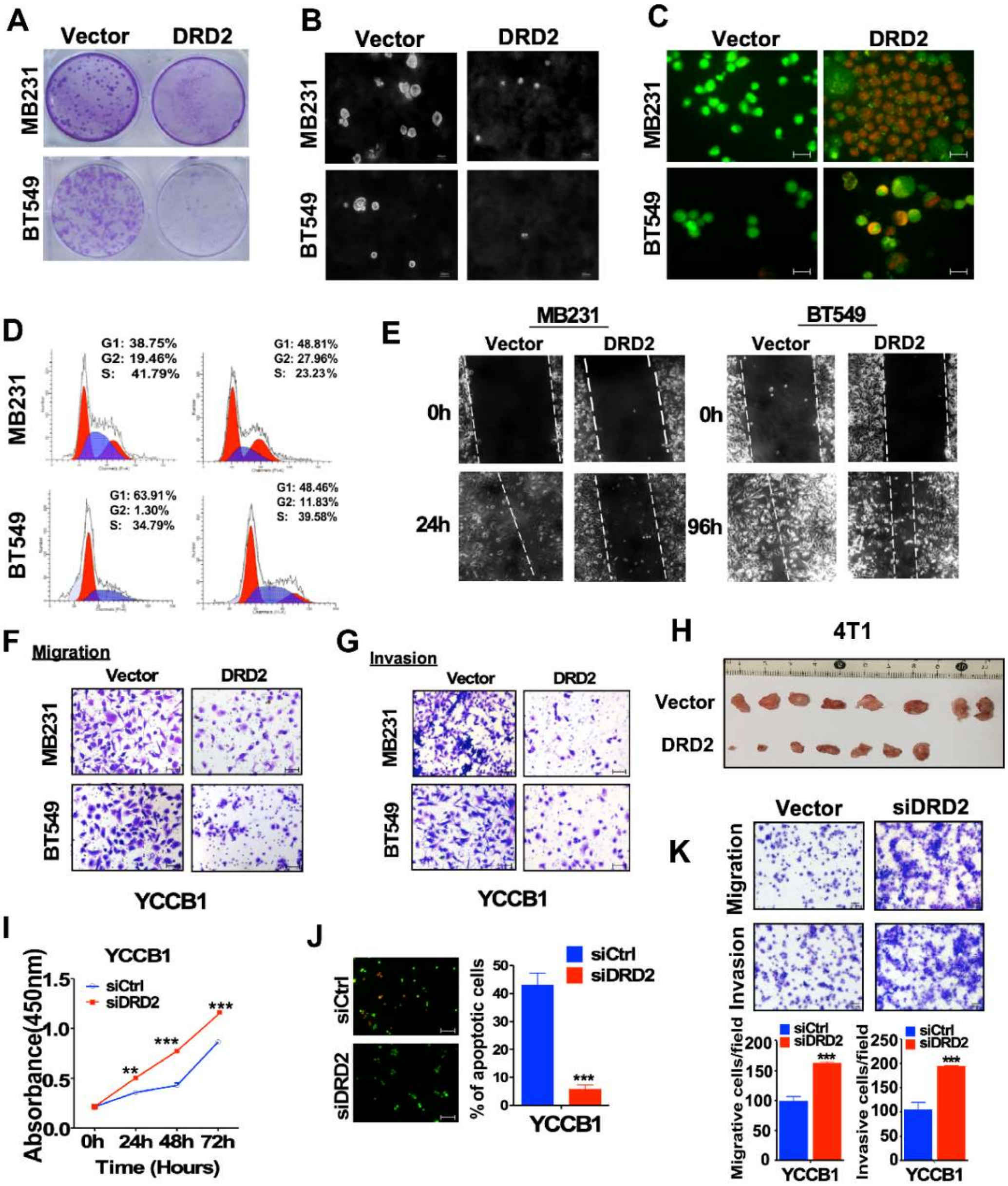

Figure S2. DRD2 inhibits progression of BrCa *in vitro and in vivo*. (**A** and **B**) Imaging of DRD2's effects on colony formation assay (A) and agar formation assay (B). (**C** and **D**) Imaging of AO/EB apoptosis assay (C) and representative flow cytometry plots of cell cycle arrest (D) in Vector- and DRD2-transfected BrCa cells. (**E**, **F** and **G**) Imaging of wound healing (E) and Transwell® coated without (F) or with (G) Matrigel in Vector- and DRD2-transfected BrCa cells. (**H**) Imaging of tumors derived from mice model. DRD2-expressed 4T1 cells were injected subcutaneously into the lower backs of BALB/c mice (8 mice per group). (**I** and **J**) Effects of downregulated DRD2 expression on proliferation accessed by CCK8 (I) and apoptosis determined by AO/EB (J) in YCCB1. (**K**) Effect of downregulated DRD2 expression on metastatic abilities determined by Transwell® assays without (upper) and with (lower) Matrigel in YCCB1. BrCa cells without treatment were used as controls. Data are presented as mean ± SD; P-value was calculated using two-tailed Student's t test. **, $p < 0.01$; ***, $p < 0.001$.

Figure S3.

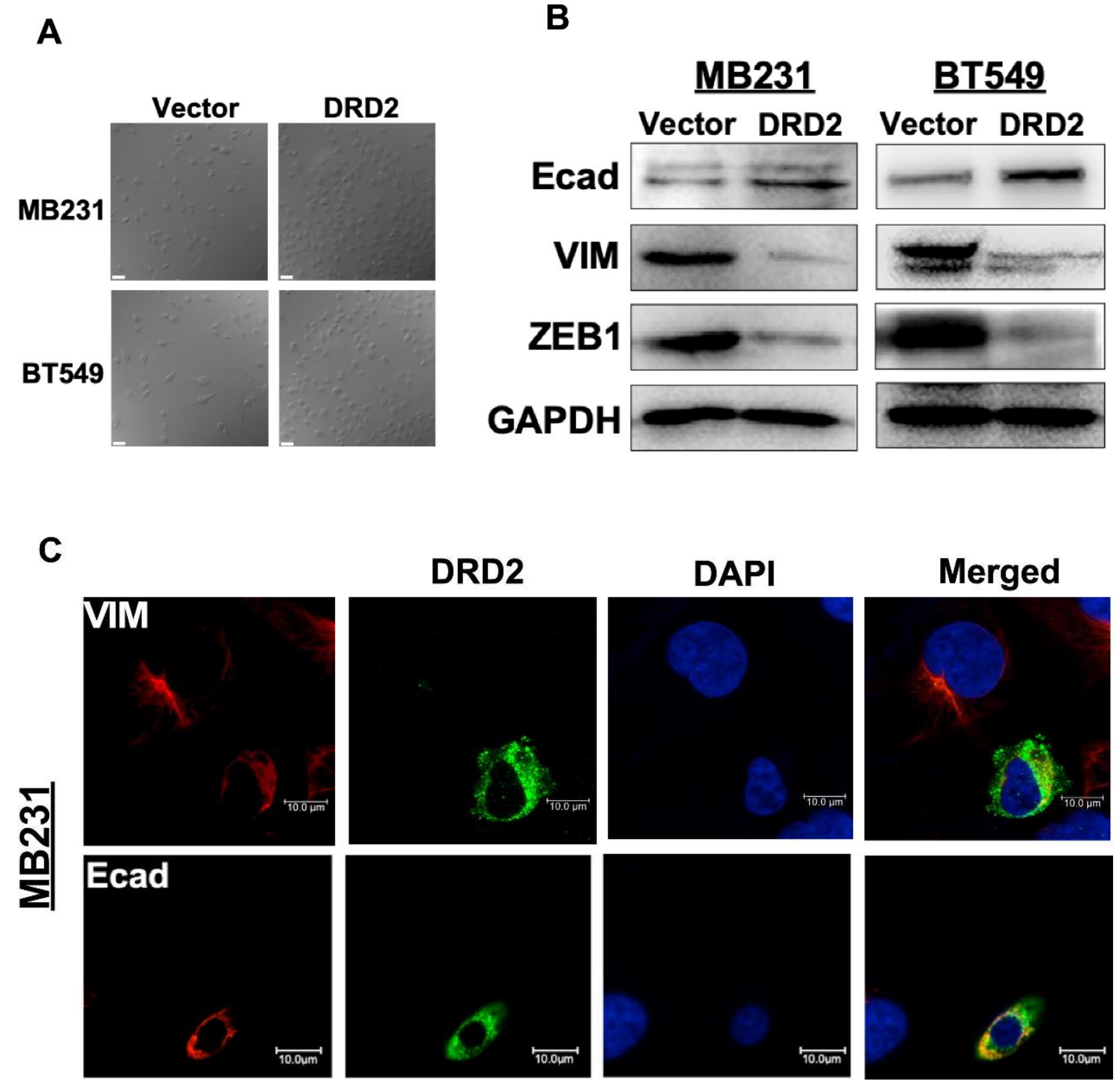

Figure S3. DRD2 suppresses EMT of BrCa cells. (A) Confocal microscopy showed morphology of BrCa cells without (left) or with (right) DRD2 expression in bright field imaging in both MDA-MB231 and BT549. Bars, 50 µm. (B) WB was used to determine the effects of DRD2 on epithelial markers (E-cadherin) and mesenchymal marker (Vimentin) as well as pro-EMT transcription factor (ZEB1) in BrCa cells. Vector-transfected BrCa cells were used as controls. (**C**) IF staining showed effects of ectopic DRD2 expression on Vimentin and E-cadherin in MDA-MB231. Nuclei were stained with DAPI. Bars, 10 µm. Vector-transfected BrCa cells were used as controls. Nuclei were stained with DAPI. Bars, 10 µm.

Figure S4.

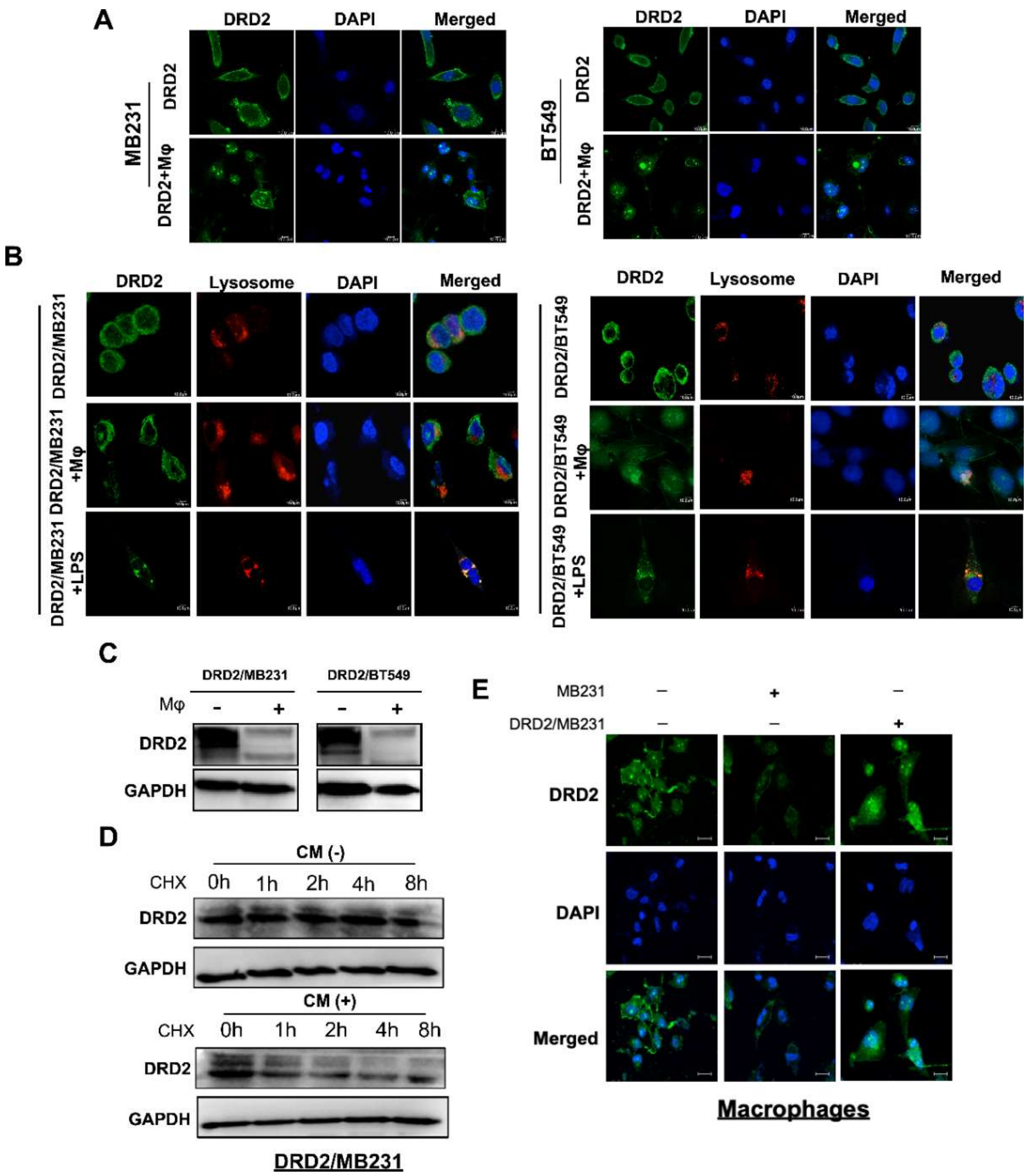

Figure S4. DRD2 is induced endocytosis in the presence of non-selective ligands. (**A**) Representative IF staining by confocal microscopy showed subcellular location of DRD2 (green) in BrCa cells with or without Mφ stimulation. (**B**) DRD2-transfected BrCa cells co-cultured with Mφ or treated with LPS were stained with DRD2 (green) and lysosome tracker (red). And DRD2-expressing tumor cells without any treatment were used as controls. Nuclei were stained with DAPI. Bars, 10μm. (**C**) Detection of protein expression of DRD2 in BrCa cells co-cultured with Mφ. (**D**) Determination of protein stability of DRD2 by Mφ. CHX (50 μg/ml, Sigma-Aldrich) was used to inhibit synthesis of protein. DRD2/MB231 was treated with conditioned medium (CM) from upper chamber of co-cultured system and then harvested after CHX treatment at 0, 1, 2, 4, 8 h. GAPDH was used for protein integrity. (**E**) IF staining of DRD2 (green) in Mφ co-cultured with BrCa cells. And primary THP1-derived Mφ was used as control. Data are representative of three independent experiments. Nuclei were stained with DAPI. Bars, 10 μm.

Figure S5.

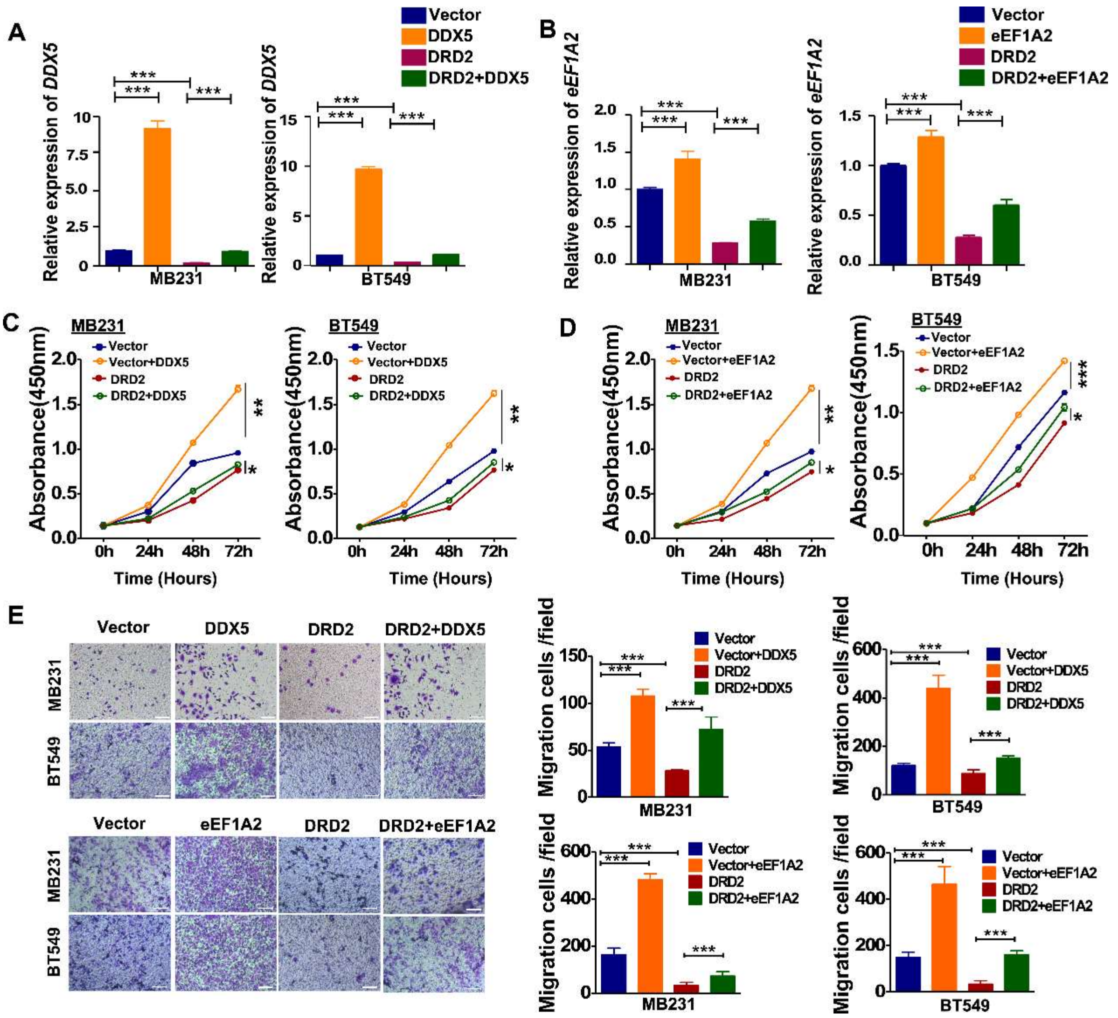

Figure S5. DRD2 suppresses proliferation and metastasis via downregulating DDX5 and eEF1A2. (**A** and **B**) Confirmation of ectopic expression. Ectopic expression of DDX5 (A) and eEF1A2 (B) were detected by qRT-PCR in MDA-MB231 and BT549. (**C**, **D** and **E**) Effects of ectopic expression of DDX5 and eEF1A2 on proliferation and metastasis were analyzed by CCK8 (C, DDX5; D, eEF1A2) and Transwell® assays in MDA-MB231 and BT549. Bars, 100 μm. Analysis of metastasis was on the right side. Data are presented as mean ± SD. P-value was calculated using two-tailed Student's t test. *, $p < 0.05$; **, $p < 0.01$; ***, $p < 0.001$.
